# Magneto-Dielectric Synergy and Multiscale Hierarchical Structure Design Enable Flexible Multipurpose Microwave Absorption and Infrared Stealth Compatibility

**DOI:** 10.1007/s40820-024-01549-4

**Published:** 2024-10-16

**Authors:** Chen Li, Leilei Liang, Baoshan Zhang, Yi Yang, Guangbin Ji

**Affiliations:** 1 School of Electronic Science and Engineering, Nanjing University, Nanjing, 210093 People’s Republic of China; 2 College of Materials Science and Technology, Nanjing University of Aeronautics and Astronautics, Nanjing, 210016 People’s Republic of China

**Keywords:** Microwave absorption, Radar-infrared compatible stealth, Wrinkled MXene, Magneto-dielectric synergy, Multifunction

## Abstract

Developing advanced stealth devices to cope with radar-infrared (IR) fusion detection and diverse application scenarios is increasingly demanded, which faces significant challenges due to conflicting microwave and IR cloaking mechanisms and functional integration limitations. Here, we propose a multiscale hierarchical structure design, integrating wrinkled MXene IR shielding layer and flexible Fe_3_O_4_@C/PDMS microwave absorption layer. The top wrinkled MXene layer induces the intensive diffuse reflection effect, shielding IR radiation signals while allowing microwave to pass through. Meanwhile, the permeable microwaves are assimilated into the bottom Fe_3_O_4_@C/PDMS layer via strong magneto-electric synergy. Through theoretical and experimental optimization, the assembled stealth devices realize a near-perfect stealth capability in both X-band (8–12 GHz) and long-wave infrared (8–14 µm) wavelength ranges. Specifically, it delivers a radar cross-section reduction of − 20 dB m^2^, a large apparent temperature modulation range (ΔT = 70 °C), and a low average IR emissivity of 0.35. Additionally, the optimal device demonstrates exceptional curved surface conformability, self-cleaning capability (contact angle ≈ 129°), and abrasion resistance (recovery time ≈ 5 s). This design strategy promotes the development of multispectral stealth technology and reinforces its applicability and durability in complex and hostile environments.

**Supplementary Information:**

The online version contains supplementary material available at 10.1007/s40820-024-01549-4.

## Highlights

- A multiscale hierarchical structure design, integrating wrinkled MXene radar-infrared shielding layer and flexible Fe_3_O_4_@C/PDMS microwave absorption layer
- The assembled stealth devices realize a near-perfect stealth capability in both X-band (8-12 GHz) and long-wave infrared (8-14 µm) wavelength ranges.
- The optimal device demonstrates exceptional curved surface conformability, self-cleaning capability (contact angle ≈ 129°), and abrasion resistance (recovery time ≈ 5 s).

**Supplementary Information:**

The online version contains supplementary material available at 10.1007/s40820-024-01549-4.

## Graphical abstract

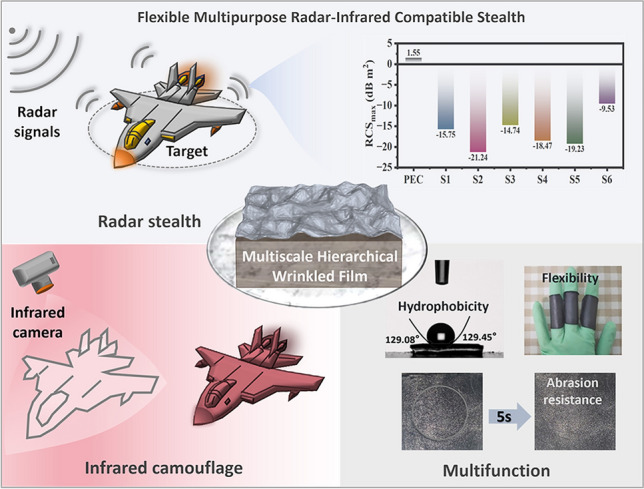

## Introduction

Continuous innovation and development in various military reconnaissance methods is driving the expansion of electronic surveillance equipment into the broader electromagnetic (EM) spectrum from visible to microwave ranges [1, 2]. The emergence of multispectral compatible stealth technology, especially radar-infrared (IR) compatible stealth, has enabled military equipment to evade dangerous and seamless reconnaissance signals at over-the-horizon ranges, thereby greatly improving their survivability and combat effectiveness [3]. Nevertheless, there is a conflict between the two camouflage mechanisms in terms of EM characterization requirements. Radar stealth requires low reflection and high absorption, while IR stealth requires high reflection and low radiation, which poses a significant challenge for material and structure design [4, 5]. In general, the prevailing strategies for compatibility camouflage typically involve the assembly of composite materials, the establishment of multi-layer film response mechanisms, and the design of metamaterial structures [6–8]. Multiscale hierarchical structure design can leverage different scale structures to interact with electromagnetic waves (EMWs) at specific wavelengths, and then integrate multi-band EM responses in a single device to achieve radar-IR compatible stealth. In practice, stealth devices also need to have additional functionality to meet some complex and extreme operating environments, such as flexibility to fit on the curved surfaces in the core components, superhydrophobicity to hinder liquid infiltration to achieve self-cleaning, and abrasion resistance to enhance service life. Indeed, exploring radar-IR) compatible stealth devices integrating multiple functional characteristics is in high demand and remains a daunting task.

Generally, the Maxwell–Wagner polarization effect, resonance behavior, or magneto-electric synergy serve to heighten microwave absorption (MWA) [9, 10]. Our group has reported that ultra-wideband absorption can be achieved by utilizing anion defect engineering to induce multipolarization effects or leveraging the pneumatic matrix to enhance multiple resonance interactions [11, 12]. Recent research on the enhancement of high-frequency multi-domain magnetic response by atomic restructuration in limited dimensions [13] and the improvement of space charge polarization loss by Built-in Electric Field (BIEF) [14] has once again supported this perspective. Among numerous magnetic/dielectric materials, the core–shell structured Fe_3_O_4_@C has emerged as a strong candidate for microwave absorbers due to its high saturation magnetization, excellent synergy, remarkable magnetic loss, cost-effectiveness, and non-toxic nature [15, 16]. Additionally, a variety of microstructures, including sea-urchin-like [17], porous X-shaped [18], nanoring [19], capsule-like [20], and helical configurations [21] provide distinctive dielectric and magnetic response characteristics for tunable and designable radar cloaking capabilities. Based on the Kittel equation ($f_{r} = \gamma H_{k} /2\pi$) [22], the introduction of additional anisotropy fields (*H*_*k*_) through geometric shape control facilitates low-frequency MWA performance of Fe_3_O_4_@C. For example, the hollow nanocubes [23] and three-dimensional foam [24] manifest their outstanding MWA performance from 2 to 8 GHz. While modulating Fe_3_O_4_@C enables high-quality radar absorbing devices, its potential for IR stealth is uncertain.

Given the IR stealth mechanism, especially in the long-wavelength infrared (LIR, 8–14 µm), manipulating IR radiation signatures can be divided into two categories: changing target temperature and adjusting surface emissivity [25, 26]. Currently, a wide range of materials (e.g., metals [27], semiconductors [28], conductive polymers [29], and phase-change materials [30]) and structures (e.g., photonic crystals [31], metamaterials [32], and plasmonic structures [33]) have been employed to regulate the IR radiation characteristics. Among these, the atomically thin two-dimensional crystal MXene stands out due to its inherent low IR emissivity comparable to that of metals, high mechanical strength, abundant terminal groups, and outstanding photothermal effects [34–37], demonstrating tremendous potential for IR stealth. Recently, MXene-based sandwich-like composites and flexible textiles can afford low emissivity surfaces for prolonged IR stealth and thermal camouflage of targets in diverse outdoor weather conditions [38, 39]. However, flat low emissivity surfaces may present high specular reflection, making targets conspicuous in IR thermography. Inspired by low-emissivity Lambertian surfaces [40], diffuse reflection can be utilized to compensate for specular reflection by scattering the radiative signal from external heat sources in all directions [41]. The feature size of wrinkled MXene layer matches the IR wavelength range very well, allowing for convenient diffuse reflection of the IR radiation signal. While low emissivity provides better camouflage for high-temperature targets. Significantly, the microscale wrinkles do not impede the transmission of radar signals. In summary, the amalgamation of microscale wrinkled MXene surfaces with Fe_3_O_4_@C nanocomposites (NPs) offers unlimited possibilities to fully utilize their respective advantages and achieve radar-IR compatible stealth in diverse application scenarios. However, the integration of materials and structures with different scale features remains a thought-provoking conundrum.

Herein, we fused diverse morphological core–shell Fe_3_O_4_@C NPs into polydimethylsiloxane (PDMS), which are then assembled with microscopically wrinkled MXene layers to construct macroscopic magnetic composite films (MCFs). In comparison with existing compatible stealth solutions, the most salient feature is that the wrinkled surface visibly weakens the IR radiation while avoiding high reflection of the radar signal. Regulating the microscale morphology of Fe_3_O_4_@C can induce anisotropy, which adjusts the lattice structure and EM parameters, thereby promoting the low-frequency MWA capability. The integrated multiscale hierarchical architecture endows the wrinkled MCFs with remarkable radar-IR compatible stealth capabilities, especially pronounced in the realm of X-band radar and LIR waves. Furthermore, the MCF demonstrates splendid flexibility, hydrophobicity, and abrasion resistance, offering broad prospects for countering multispectral surveillance in complicated environments.

## Experimental Section

### Materials

Hexahydrated iron (III) chloride (FeCl_3_·6H_2_O), sodium hydroxide (NaOH), sodium sulfate (Na_2_SO_4_), and dopamine hydrochloride (C_8_H_12_ClNO_2_) were purchased from National Pharmaceutical Group Chemical Reagent Co., Ltd. Tris–HCl buffer (10 mM, pH = 8.5) was procured from Shanghai Yuanye Biotechnology Co., Ltd. Polydimethylsiloxane (PDMS) was obtained from Dow Corning Corporation. MXene (5 mg mL^−1^) and dielectric elastomer (DE, VHB 4905) were sourced from 3A Chemical Corporation. Ethanol (C_2_H_5_OH, 99.7%) and deionized (DI) water were purchased from Nanjing Chemical Reagent Co., Ltd.

### Synthesis of Fe_3_O_4_@C Nanocomposites

Initially, 27.03 g of FeCl_3_·6H_2_O (2 M) and 10.8 g of NaOH (5.4 M) were separately dissolved in 50 mL of DI water and sonicated until homogeneous solutions were formed. Subsequently, the solutions were combined and vigorously stirred for 5 min in a water bath at 75 °C. This was followed by the preparation of various Na_2_SO_4_ solutions (0.0, 0.6, and 1.2 M) which were added to the mixed solution with continuous stirring until the appearance of a gel-like red precipitate. The precipitate was then transferred to a 100 mL high-pressure reactor and aged in an oven at 100 °C for 4 days to yield a brick-red product. The product was washed multiple times through centrifugation using C_2_H_5_OH and DI water solutions. Lastly, the various morphologies of Fe_2_O_3_ were attained by drying the product overnight at 60 °C.

For the prepared Fe_2_O_3_ samples with concentrations of 0.0, 0.6, and 1.2 M, 350 mg was extracted and combined with 200 mL of Tris–HCl buffer under sonication for 10 min. Subsequently, 160 mg of C_8_H_12_ClNO_2_ was added, and the mixture was magnetically stirred for 24 h. The resulting solution was vacuum-filtered, dried, and annealed at 500 °C in an Ar atmosphere for 4 h. The final black powdered product obtained was Fe_3_O_4_@C (0.0, 0.6, and 1.2 M).

### Preparation of Flexible Fe_3_O_4_@C/PDMS Magnetic Films

The differently shaped Fe_3_O_4_@C samples prepared at various concentrations (0.0, 0.6, and 1.2 M) were combined in mass ratios of 1:3:2, 2:1:3, and 3:2:1, respectively. Following this, the mixed Fe_3_O_4_@C samples were blended with PDMS in a 2:1 ratio and thoroughly stirred to produce a viscous colloidal mixture. The resulting colloidal mixture was then carefully poured into a polytetrafluoroethylene concave mold measuring 4 × 4 cm^2^ and flattened using a glass slide. Subsequently, the Fe_3_O_4_@C/PDMS magnetic film was acquired by drying the mixture in an oven set at 60 °C and demolding the film post-drying.

### Preparation of Hierarchical Wrinkled Magnetic Composite Films

Firstly, prepare a 2 mg mL^−1^ MXene solution by diluting the few-layer MXene dispersion (5 mg mL^−1^) in anhydrous ethanol (C_2_H_5_OH, 99.7%). Subsequently, pre-stretch the dielectric elastomer (DE) to a strain of 160% and press a mask with an inner diameter of 6.5 × 6.5 cm^2^ onto the pre-stretched DE. Following this, employ a sprayer (Schneider Electric, S-13) to spray the MXene solution inside the mask. After drying, the mask should be removed, and the DE coated with MXene should retract to its original position. Surface stress instability during the retraction process of the elastomer can create surface wrinkling patterns. Finally, affix the prepared wrinkled MXene layer onto the flexible Fe_3_O_4_@C/PDMS magnetic film.

### Characterization

The phase composition and structural stability were determined by X-ray diffraction (XRD, model Smartlab 9), X-ray photoelectron spectroscope (XPS, model Escalab 250Xi), Raman spectrometer (Raman, model LabRAM HR Evolution), and thermal gravimetric analyzer (TG, model NETZSCH STA 449F3). The microstructure and morphology were characterized using a scanning electron microscope (SEM, model Apreo 2) and a transmission electron microscope (TEM, model Talos F200X). Experimental setups and film performance were photographed and filmed using a smartphone camera (Vivo X100, China). The magnetic properties were investigated using a vibrating sample magnetometer (VSM, model MPMS). Dynamic infrared emissivity spectra were measured using a Fourier-transform infrared spectrometer (FTIR, Tensor 27, Bruker, Germany) and referenced against a blackbody radiation source (DY-HT1, D-MEI Instruments, China). Real-time infrared images, videos, and apparent temperatures were captured using a Fotric 227 s infrared camera. Electromagnetic parameters were measured using a vector network analyzer (VNA, Agilent E8363B, USA) employing coaxial methods.

## Results and Discussion

### Fabrication and Characterization of Multiscale Hierarchical Wrinkled MCFs

**Fig. 1:**
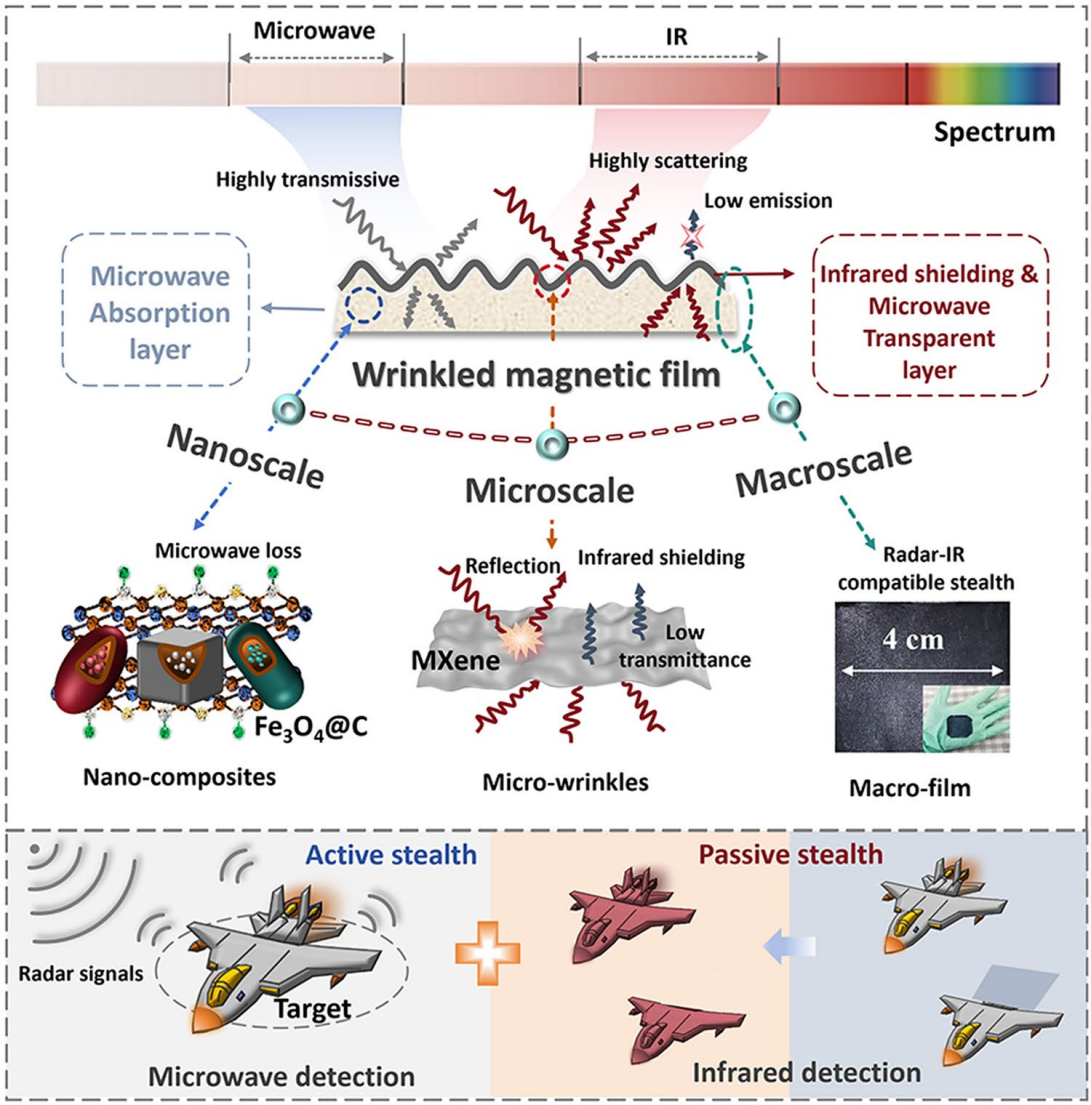
Design concept of multiscale hierarchical wrinkled MCFs and their radar-IR compatible stealth applications

**Fig. 2:**
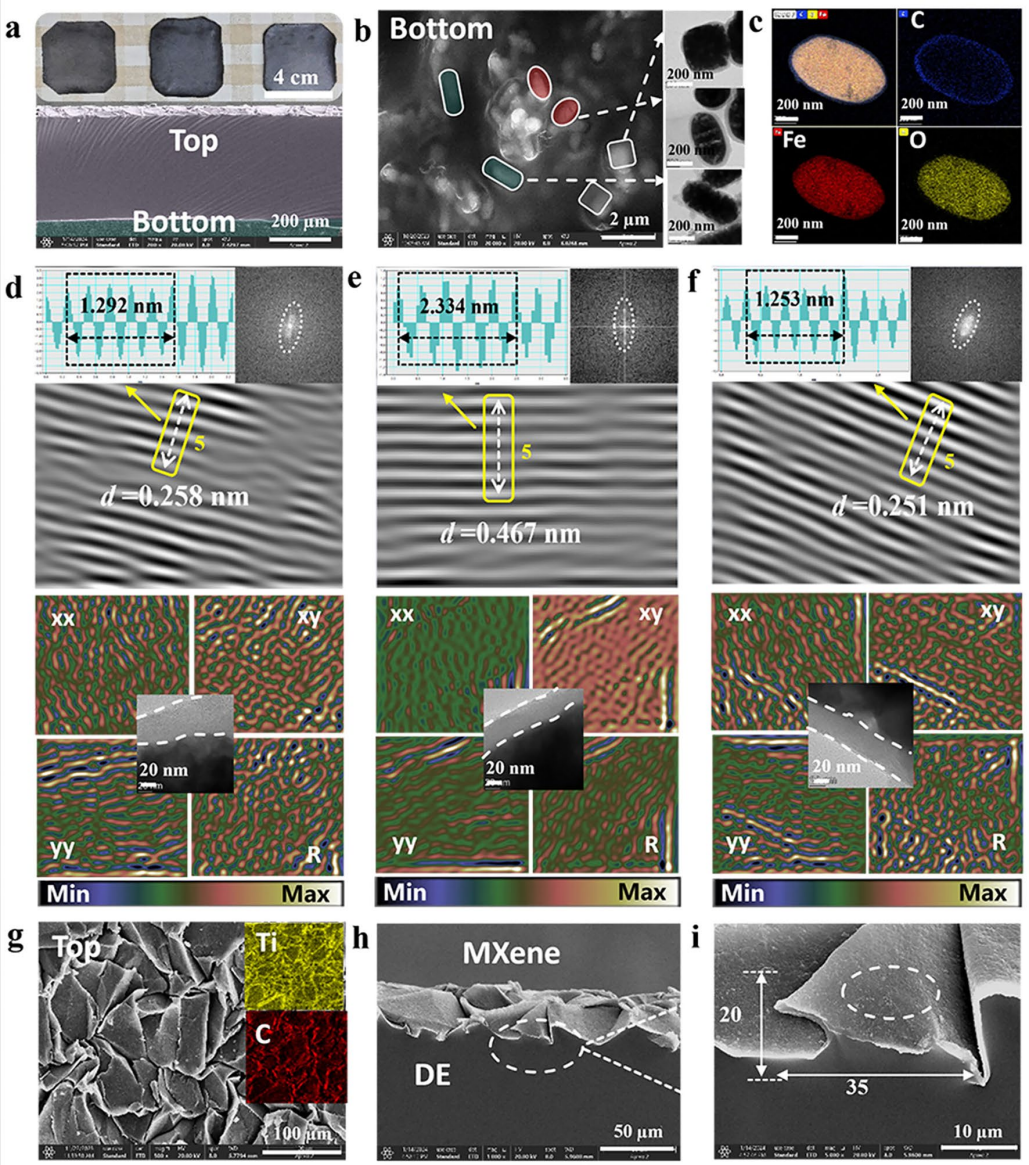
Morphological characterization of wrinkled MCFs.** a** Cross-sectional SEM images and digital photographs of MCFs. **b** Typical SEM and TEM images of Fe_3_O_4_@C/PDMS. **c** HAADF-STEM and element mapping images for ellipsoidal (0.6 M) Fe_3_O_4_@C. Inverse fast Fourier transformation, typical TEM images and (GPA) patterns of **d** cubic (0.0 M), **e** ellipsoidal (0.6 M), and **f** peanut-shaped (1.2 M) Fe_3_O_4_@C. **g** Typical SEM and EDS images of wrinkled MXene. **h, i** Cross-sectional SEM images of wrinkled MXene layer

The designed multiscale hierarchical MCF consists of the top IR shielding layer and the bottom MWA layer (Fig. 1). At the nanoscale, the core–shell Fe_3_O_4_@C NPs introduce significant dielectric and magnetic losses to attenuate incoming radar signals. At the microscale, the wrinkled MXene structure with an intrinsically high refractive index (*n*) reflects IR radiation signals and minimizes emissivity. At the macroscale, the integration of centimeter-level film heightens the camouflage capability of military equipment in response to radar-IR fuse detection and tracking. A clear assembly process of the multiscale hierarchical wrinkled MCF is depicted in Fig. S1. Initially, to fabricate the bottom film, the core–shell Fe_3_O_4_@C prepared in Fig. S2 was vigorously blended with PDMS and then uniformly applied through blade coating. Subsequently, the dielectric elastomer (DE) was pre-stretched and sprayed with the Ti_3_C_2_T_x_ MXene solution, followed by the mechanical contraction of the DE to attain a wrinkled MXene layer. Lastly, adhere the wrinkled MXene layer onto the flexible Fe_3_O_4_@C/PDMS film. Digital images of macroscopic wrinkled MCFs and their hierarchical structure are displayed in Fig. 2a. The bottom layer is composed of aggregated cubic, ellipsoidal, and peanut-shaped NPs, surrounded by PDMS (Figs. 2b and S3). Aggregation occurs due to the high viscosity of PDMS and the inherent tendency of nanomaterials to aggregate when dispersed in liquid. The morphological variation (Fig. S4) is controlled by the Na_2_SO_4_ concentration (0.0, 0.6, and 1.2 M), which is thermodynamically driven by adjusting the surface energy of different crystal planes [42]. Eventually, the crystal planes with faster growth rates gradually disappear, while those with slower growth rates are retained. XRD analysis shows that all three morphological NPs correspond to Fe_2_O_3_ (89–0596), without impurities (Fig. S5). Core–shell NPs constructed using Fe_2_O_3_ templates (Figs. S6 and S7) have the light-colored outer layer wrapped around the dark-colored inner layer, with internal voids that may originate from high-temperature annealing.

In the case of ellipsoid micromorphology, the outer layer of the core–shell consists of C elements, while the inner layer contains abundant Fe and O elements (Fig. 2c and Table S1). The corresponding diffraction peaks confirm the presence of Fe_3_O_4_, but the carbon peak cannot be observed in the XRD (Fig. S8a). The Raman spectrum in Fig. S8b exhibits strong peaks at 1340 cm^−1^ (D band) and 1596 cm^−1^ (G band), validating the formation of the carbon layer. The increase in the *I*_D_/*I*_G_ ratio from 0.85 to 0.96 indicates an elevated level of defects, which may reduce the electrical conductivity of the carbon layer [43]. Furthermore, the TGA indicates that the sample does not experience apparent weight loss (< 20%), demonstrating the relatively high thermal stability of Fe_3_O_4_@C (Fig. S9) [17]. Subsequent XPS analysis delves deeper into the surface structure of Fe_3_O_4_@C (Figs. S10–S12). The more detailed lattice structure analysis of Fe_3_O_4_@C is depicted in Fig. 2d–f, where the internal strain effects were investigated using inverse fast Fourier transform (IFFT) and geometric phase analysis (GPA) techniques [44]. The reasons for 0.0 and 1.2 M structural damage (Fig. S7) prompt an investigation into lattice strain [45]. Based on the Gibbs–Thomson equation, NPs tend to compress their size to achieve more stable state and reduce surface energy, inevitably leading to strain effects [46]. By observing the lattice fringes of Fe_3_O_4_@C in the HTEM images via IFFT, the lattice spacing (*d*) for the 0.0, 0.6, and 1.2 M was measured to be 0.258, 0.467, and 0.251 nm, respectively. The decreased *d* value confirms an increased degree of lattice strain, which explains the morphological damage observed in the 0.0 and 1.2 M. The GPA images further present relatively uniform stress within the core–shell structure, particularly underling the stress enhancement at the interface. The top layer displays the densely wrinkled morphology, and EDS analysis showcases the distribution of Ti, C, O, and F elements across the entire wrinkled structure (Figs. 2g and S13a, b). The elemental composition proportions highlight Ti and C as the primary constituents (Fig. S13c), affirming the presence of Ti_3_C_2_T_x_. The cross-sectional SEM image shows the consistent distribution of wrinkled MXene on the upper layer of DE, with individual wrinkles of approximately 35 μm in width and 20 μm in height (Fig. 2h, i). Consequently, the multiscale hierarchical MCF consisting of bottom flexible Fe_3_O_4_@C/PDMS and top wrinkled MXene layer were successfully assembled.

### Modulable Fe_3_O_4_@C/PDMS for Compatible Stealth Performance

To assess the radar stealth capability, EM parameter testing was initially conducted on the Fe_3_O_4_@C NPs. The complex permittivity ($\varepsilon = \varepsilon^{\prime } - j\varepsilon^{\prime \prime }$) and complex permeability ($\mu = \mu^{\prime } - j\mu^{\prime \prime }$) determine the magnitude of *RL*, while the *RL*, effective absorption bandwidth (*EAB*), and their corresponding thickness (*d*_*m*_) directly reflect the quality of the MWA performance [47]. Figure S14a shows the variation curves of the EM parameters for 0.0, 0.6, and 1.2 M. The high $\mu^{\prime \prime }$ and low $\varepsilon^{\prime \prime }$, combined with suitable $\varepsilon^{\prime }$ and $\mu^{\prime }$, promote MWA in the low frequency range of 2–8 GHz. Particularly, 0.6 M (with the highest $\varepsilon^{\prime }$ value) has an *RL* of  −61.28 dB at 2.20 GHz, with a *d*_*m*_ of 5.79 mm. Among them, the *RL* of 0.0 and 1.2 M are − 65.04 and − 54.60 dB at 7.56 and 3.84 GHz, corresponding to *d*_*m*_ of 2.89 and 4.37 mm (Fig. S15). The prominence of Fe_3_O_4_@C in low-frequency MWA is closely linked to the coupling of dielectric and magnetic parameters, notably manifested by the negative values of $\mu^{\prime \prime }$ within the high-frequency range of 12–18 GHz. According to Maxwell’s equations, alternating electric fields generate a counter-inductive magnetic field. When the induced magnetic field surpasses the original one, magnetic energy is radiated from or converted to electric energy within the Fe_3_O_4_@C [48]. The variations of the loss tangent and Cole–Cole semicircle reveal that dielectric loss takes the lead in 12–18 GHz range, indicating the modulation of magnetic behavior by dielectric behavior (Fig. S16a–c and g–i). According to the Debye theory, the $\varepsilon^{\prime \prime }$ values are composed of $\varepsilon_{p}^{\prime \prime }$ and $\varepsilon_{c}^{\prime \prime }$ ($\varepsilon_{c}^{\prime \prime } = \sigma /2\pi f\varepsilon_{0}$), which represent the polarization loss and conductivity loss, respectively. The low conductivity values in Fig.S17a indicate that defects in the carbon layer reduce conductivity, while a detailed analysis of the $\varepsilon_{c}^{\prime \prime } \sim f$ and $\varepsilon_{p}^{\prime \prime } \sim f$ curves in Fig. S17b, c reveals that dielectric losses are primarily contributed by polarization losses. Figure S16d–f presents that the magnetic losses mainly stem from natural resonance (2–4 GHz) and eddy current losses (4–12 GHz) [49]. Meanwhile, the hysteresis loops (*M-H*) (Fig. S15b) depict saturation magnetization strengths (*M*_*s*_) of 83.71, 83.18, and 88.08 emu g^−1^, and coercivities (*H*_*c*_) of 14.51, 15.55, and 10.19 Oe respectively. These values signify that the enhanced magnetic properties of Fe_3_O_4_@C induce substantial EMW attenuation at low-frequency range.

**Fig. 3:**
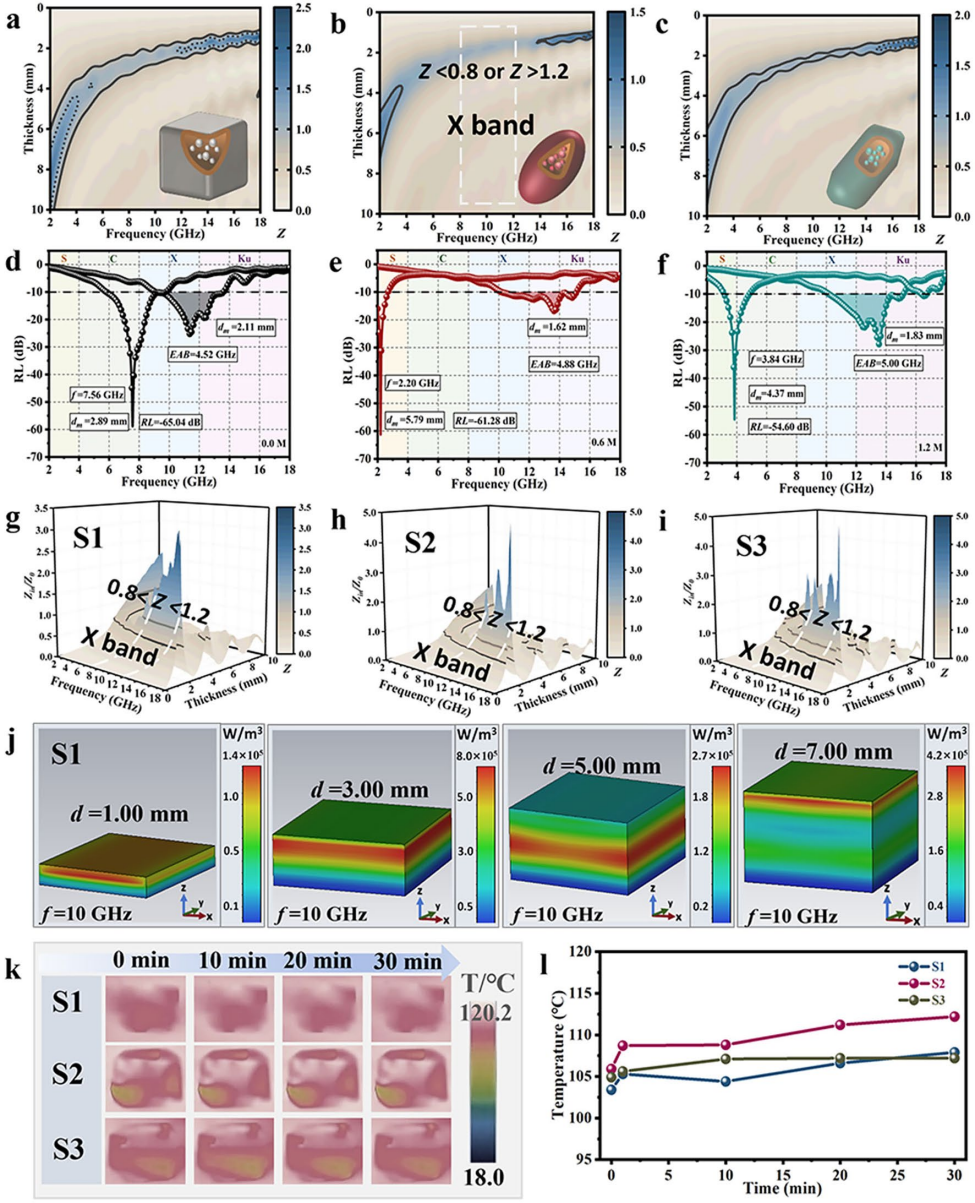
Compatible stealth performance of Fe_3_O_4_@C/PDMS. **a–c** 2D Impedance matching color maps of Fe_3_O_4_@C (0.0, 0.6, and 1.2 M. **d–f** Typical 2D curves of Fe_3_O_4_@C (0.0, 0.6, and 1.2 M). **g–i** 3D Impedance matching color maps of S1, S2, and S3. **j** Electromagnetic power loss density map of S1 with different thickness derived from CST at 10 GHz. **k** Thermal IR images of S1–S3 at different heating times (0, 10, 20, and 30 min). **l** Apparent temperature curve for S1–S3 during 30 min

Furthermore, Fig. 3d–f illustrates that three samples reach optimal *EAB* exceeding 4.50 GHz, demonstrating robust low-frequency MWA performance and extensive *EAB*. According to transmission line theory, impedance (*Z*) matching serves as a prerequisite for the occurrence of MWA phenomena [50–52]. Unfortunately, although Fe_3_O_4_@C has a favorable attenuation constant ($\alpha$) (Fig. S18), 0.6 M exhibits an impedance mismatch in the X-band (Fig. 3a–c). This mismatch arises from the unsuitability of the material’s dielectric constant and magnetic permeability within this specific frequency range. The EM parameters were adjusted through a mixed modulation process to compensate for the lack of impedance matching. The flexible Fe_3_O_4_@C/PDMS films were prepared with mixing ratios of 1:3:2, 2:1:3, and 3:2:1, labeled as S1, S2, and S3, respectively. Undoubtedly, S1–S3 proclaim decent MWA and satisfactory *Z* in the X-band (Figs. 3g–i and S19), which imply the validity of the adjustments. The optimal *EAB* for the S2 (4.34 GHz) covers the X-band, while the *RL* reaches − 60.62 dB at 4.74 GHz. To further dissect the influence of MWA layer thickness on absorption performance, we actualized CST simulations of the S1, S2, and S3. Thicknesses of 1, 3, 5, and 7 mm were chosen, and EM power loss density was compared. It is evident that the EM power loss density at 3 mm is relatively high, and the loss is mainly concentrated within the surface at a depth of 2–3 mm, irrespective of the variation in film thickness (Figs. 3j, S20, and S21). In order to certify the IR stealth capability of Fe_3_O_4_@C/PDMS, IR thermography was recorded under a heat source of 120 °C. The corresponding thermal IR images show that the Fe_3_O_4_@C/PDMS films have lousy IR stealth capability (Fig. 3k). The thermal conductivity of the Fe_3_O_4_@C/PDMS film (0.2825 W m^−1^ K^−1^) also reflects that its thermal insulation performance is not ideal (Table S2). Figure 3l illustrates the apparent temperature variation profile of the film over a period of 30 min, implying stability.

### Enhanced Compatible Stealth Performance by Accessorial Wrinkled MXene Layer

**Fig. 4:**
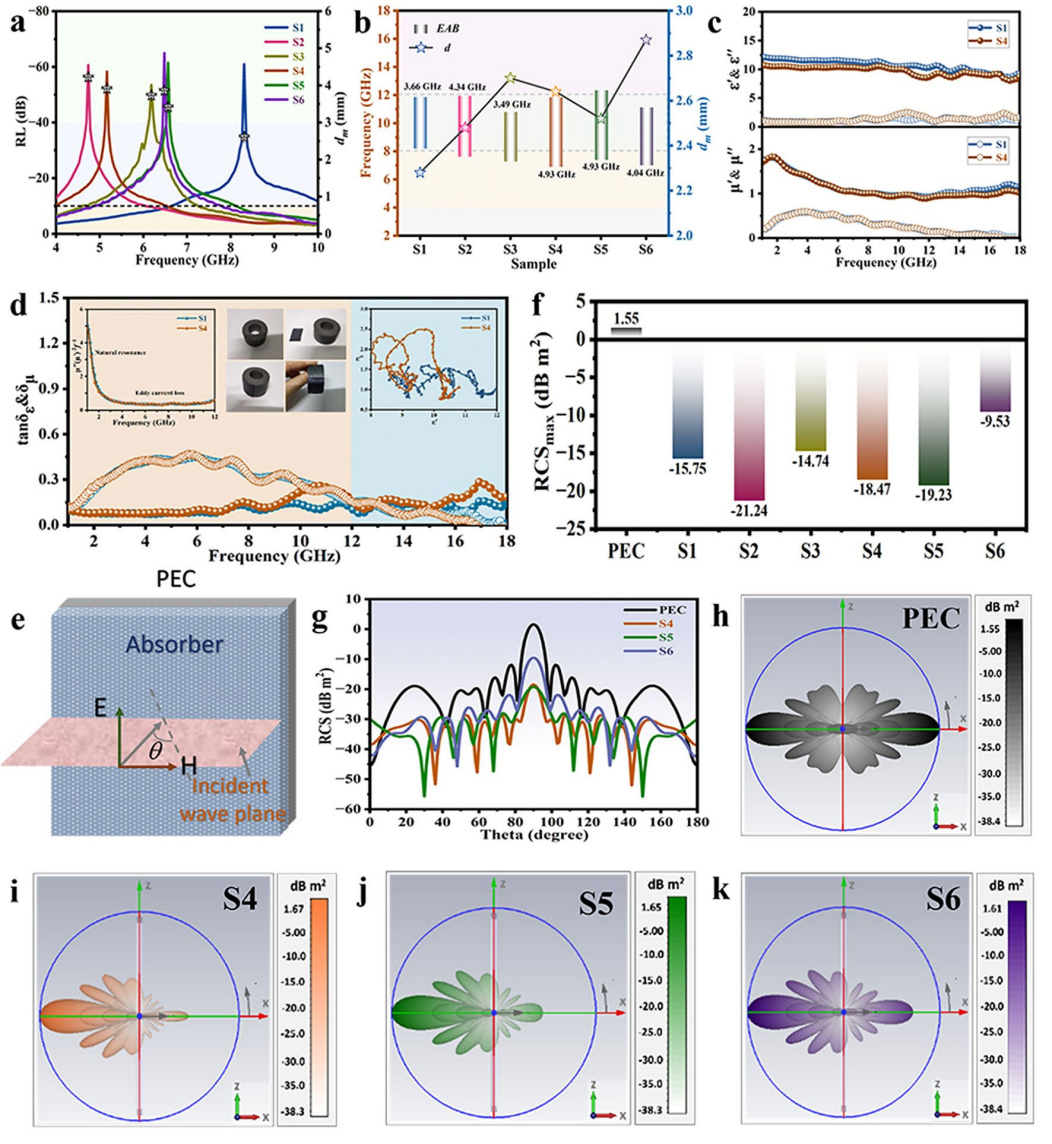
Radar stealth performance of wrinkled MCFs. **a** *RL*-*f* statistic curves for S1–S6. **b** Optimal *EAB* columnar statistics for S1-S6. **c** Complex permittivity and permeability of S1 and S4. **d** Dielectric and magnetic loss tangent, eddy current curve, display on film magnetism, and Cole–Cole plots of S1 and S4. **e** Schematic diagram of CST simulation. **f** Statistical graph of RCS_max_. **g** RCS simulated curves of PEC, S4, S5, and S6. **h–k** 3D radar wave scattering signals of PEC, S4, S5, and S6

Flexible Fe_3_O_4_@C/PDMS with accessorial wrinkled MXene layers on the surface were labeled as S4, S5, and S6. To investigate the radar stealth capability of the hierarchical wrinkled MCF, statistics on the maximum *RL* and corresponding *f* and *d*_*m*_, as well as the optimal *EAB* and corresponding *f* range of S1-S6, are presented in Fig. 4a, b. Overall, the most prominent absorption peaks of the samples occur within the range of 4–10 GHz, with the characteristic of low-frequency absorption remaining intact (Figs. S22 and S23). Particularly, the *EAB* covers nearly the entire X-band, which suggests the microwave transparency of the wrinkled MXene layer. Figure 4c displays the EM parameter curves for S1 and S4, indicating that the MXene layer has minimal impact on $\varepsilon$ and $\mu$. To explore the underlying mechanisms of MCFs, EM parameters analysis was conducted. Taking S1 and S4 as representatives, Fig. 4d uncovers the variation curves of $\tan \delta_{\varepsilon }$ and $\tan \delta_{\mu }$, and the left inset observes that the magnetic losses are a combination of natural resonance from 1 to 4 GHz and eddy current losses from 4–12 GHz. Magnetic tests demonstrate the rapid adsorption of the MCFs by a magnet (≈ 1 s, middle inset of Fig. 4d and Video S1). Dielectric losses dominate between 12 and 18 GHz, mainly originating from multiple relaxation losses due to interfacial effects (right inset of Fig. 4d) [53]. The same analysis yields specific loss sources for S2, S5, S3, and S6 (Figs. S24–S27). To unveil the potential of the designed hierarchical wrinkled MCF in practical applications, radar cross-section (RCS) simulations were proceeded using CST. RCS characterizes the intensity of the radar echo when a target is detected by radar waves, with a weaker signal indicating better stealth capabilities of the target [54]. The plane wave incident on the established model (100 × 100 mm^2^) was monitored at varying angles of *θ* from 0° to 180° (Fig. 4e). Compared to pure perfect electric conductor (PEC), the three-dimensional RCS signals after coating absorbers S4, S5, and S6 exhibit a significant reduction (Fig. 4h–k). Moreover, the RCS two-dimensional curves at different angles and the RCS_max_ statistics chart (*θ* = 90°) show an approximate 20 dB m^2^ decrease with absorber coatings (Fig. 4f, g). The simulation results for S1, S2, and S3 also confirm the strong MWA capabilities of the designed film (Fig. S28), consistent with the actual test.

**Fig. 5:**
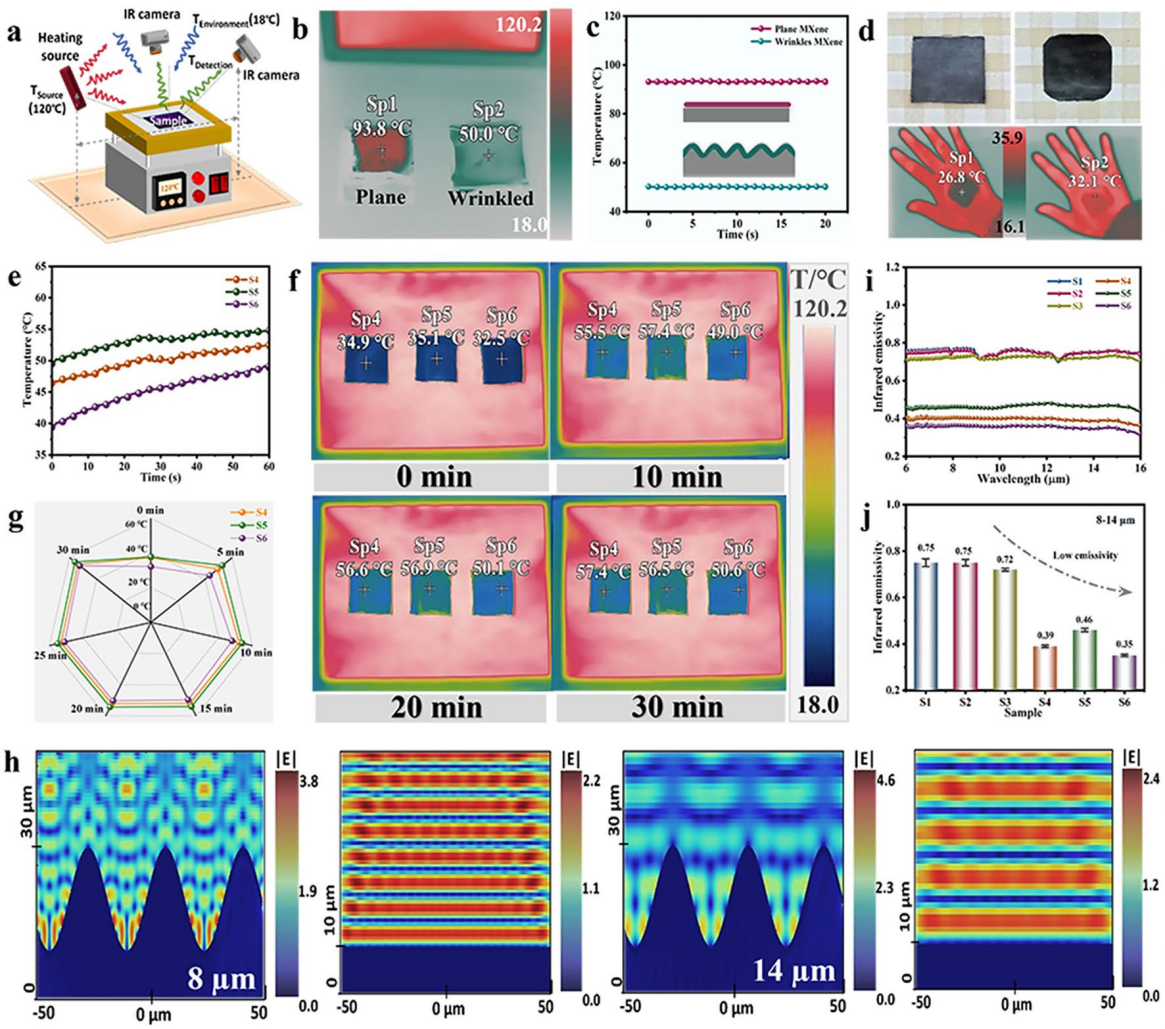
Infrared stealth performance of wrinkled MCFs. **a** Schematic of thermal IR images measurement setup at different observing angles. **b** Thermal IR images of smooth (left) and wrinkled (right) MXene MCFs. **c** Apparent temperature curve of smooth (left) and wrinkled (right) MXene MCFs. **d** Digital and thermal IR images of wrinkled MXene MCFs (left) and Fe_3_O_4_@C/PDMS films (right) at RT. **e** Apparent temperature curve of S4–S6 during 60 s. **f** Thermal IR images of S4-S6 at different heating times (0, 10, 20, and 30 min). **g** Apparent temperature statistics of S4-S6 during 0–30 min. **h** Electric field distributions 8 and 14 μm of wrinkled and smooth MCFs. **i** IR emissivity curves of S1-S6. **j** Columnar statistics of IR emissivity of S1-S6 in 8–14 µm

In practical camouflage scenarios, the thermal radiation effect of the target changes depending on the IR camera’s position (Fig. 5a). The wrinkled structure design solves the symmetrical positioning problem of the IR camera and the heat source, and prevents the camouflage target from losing its stealth capability due to specular reflections induced by external radiation signals [40]. Figure 5b and Video S2 depict the thermal camouflage capabilities of the smooth and wrinkled MXene under 120 °C heat radiation, with specific imaging setups shown in Fig. S29. Notably, the wrinkled structure demonstrates outstanding thermal camouflage effects, maintaining the apparent temperature of approximately 50.0 °C for the initial 20 s (Fig. 5c). Comparative images at 5, 10, 15, 20, 25, and 30 min in Fig. S30 present virtually unchanged radiation temperatures, underscoring the thermal stability of the MXene layer. Figure 5d primarily illustrates a comparison between physical photographs of the hierarchical wrinkled MCF and the standalone Fe_3_O_4_@C/PDMS film, captured by a mobile phone and an IR camera. The film covered with an MXene layer exhibits a lighter color (on the left), showcasing the superior insulation capacity when placed on the palm compared to the standalone Fe_3_O_4_@C/PDMS film. Thermal camouflage evaluations were conducted on all prepared MCF (under 120 °C heat source radiation). Encouragingly, the apparent temperature of S4, S5, and S6 fluctuated only slightly during the first 60 s and eventually stabilized below 55 °C (Fig. 5e). The apparent temperature variations of the films ranged from 0 to 30 min, with S4, S5, and S6 hovering around 50 °C (Figs. 5f, S31 and Video S3). Figure 5g summarizes the temperature variations of S4–S6 over a period of 30 min, showing the potential for long-term thermal camouflage. The addition of MXene layer results in the IR radiation temperature difference (ΔT) of up to 70 °C between the disguised target and the background heat source. Additionally, the real surface temperatures of MCF and Fe_3_O_4_@C/PDMS at 120 °C were measured using high-precision thermocouples for comparison with the apparent temperatures detected by IR camera (Fig. S32).

Optical simulations were performed by utilizing the material’s dielectric parameter to investigate the IR stealth impact of the wrinkle structure in hierarchical MCF (Fig. S33). The theoretical simulation model (left of Fig.S34a) highlights the crucial role of the wrinkle structure (width × height of 35 × 20 µm^2^) on tuning the IR stealth. Specifically, for different IR wavelengths in the range of 6–16 µm, the wrinkle structure manifests electric field enhancement effects between the two MXene peaks (right of Fig. S34a). This phenomenon arises from surface plasmon resonance leading to strong field confinement and localization, which effectively absorbs incident IR light [55]. However, smooth MXene structure show that the electric field forms periodic oscillations along the vertical direction (Fig. S34b). This periodic oscillation suggests that fierce interference effects occur between the IR waves reflected by the MXene layer and the incident waves, which leads to specular reflections and then poses a challenge for IR stealth. Besides, the electric field distributions reveal that the enhancement effect at 8 µm is superior to that at 14 µm, which is likely associated with the characteristic size of the MXene wrinkles (Fig. 5h). Achieving IR stealth requires altering the IR radiation characteristics of the target, including regulating apparent temperature and adjusting emissivity [56]. Through wrinkle structure simulations, the designed film demonstrates high reflectivity (0.65 ~ 0.75) and low transmittance (0 ~ 0.008) within the 6–16 µm wavelength range (Fig. S35). Preliminary theoretical predictions indicate that wrinkled MCF may possess low IR emissivity, as determined by IR spectroscopy testing (Fig. 5i). The emissivity of S1, S2, and S3 within the 6–16 µm is approximately 0.75, while samples S4, S5, and S6 exhibit a lower emissivity of around 0.35. The emissivity test results are consistent with the simulation results, and the incorporation of the wrinkled MXene layer drastically reduces the IR emissivity (Fig. 5j). This tendency is attributed to the wrinkle-induced diffuse reflection, which scatters the incident IR waves in various directions, diminishing the IR radiation signal into the device interior. Moreover, the IR radiation signals generated from the bottom are blocked by the conductive MXene layer and cannot be radiated into the environment.

### Stealth Mechanisms and Applications in Complicated Environments of Multiscale Hierarchical Wrinkled MCFs

**Fig. 6:**
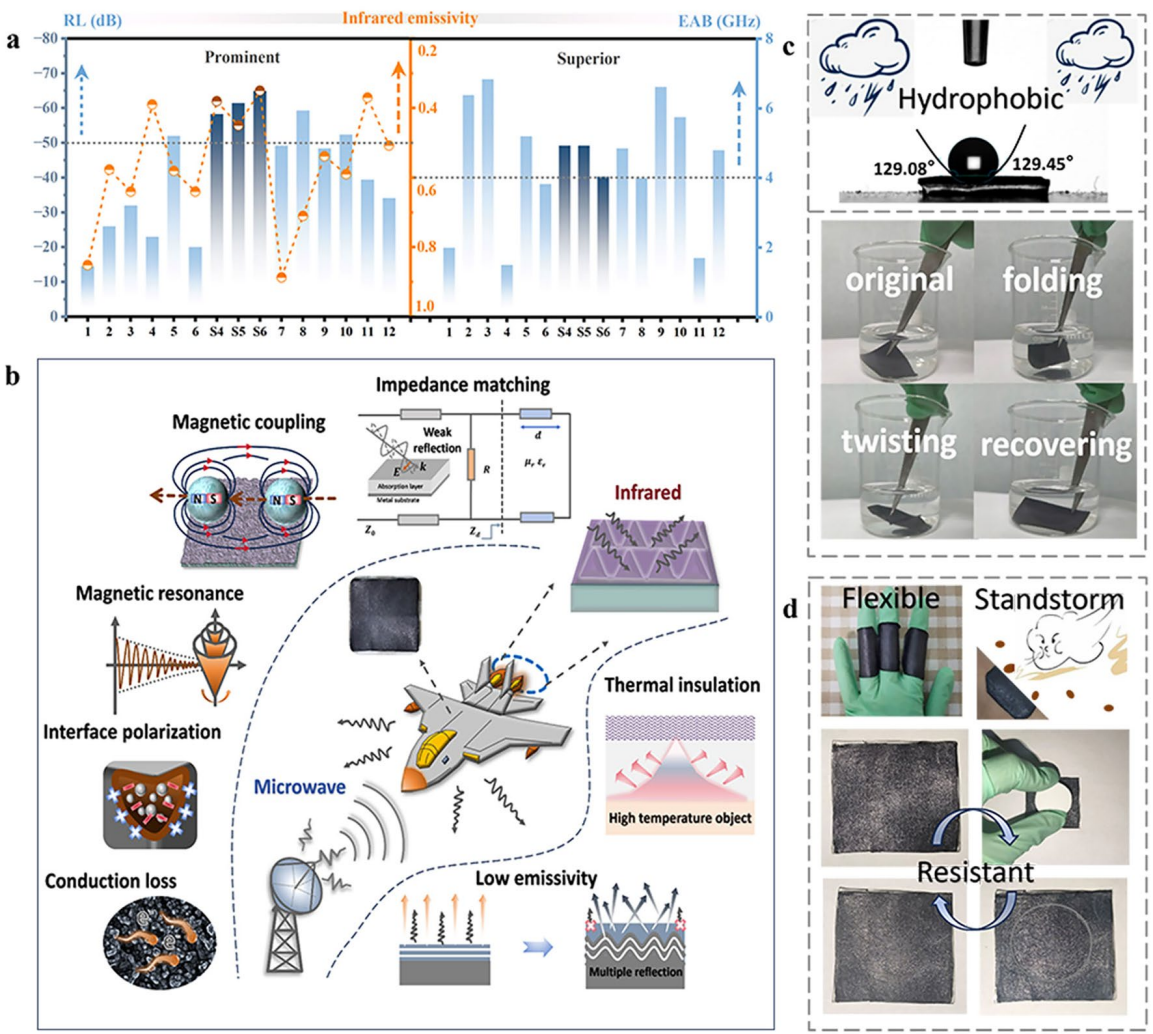
Stealth mechanisms and multifunctionality. **a** Comparative statistical chart on radar-IR compatible stealth performance. **b** Mechanisms of radar-IR compatible stealth. **c** Schematic representation of contact angle and digital images of the wrinkled MCFs in water. **d** Flexibility and abrasion resistance of the wrinkled MCFs

The challenges faced by traditional single-band camouflage in the face of multispectral coordinated detection techniques can be overcome through multiscale hierarchical structure design. In general, the wrinkled MCF compares favorably to the published research in terms of radar stealth and IR camouflage, all performing at a satisfactory level. Specifically, our work achieves *RL* consistently exceeding − 50 dB, an *EAB* exceeding 4.00 GHz, RCS reduction of 20 dB m^2^, with an IR emissivity below 0.5 and (Fig. 6a, Tables S3 and S4). Overall, the MCFs are dedicated to improving the electronic warfare capabilities of weaponry by integrating nanomaterials and microscopic wrinkles. Implementation of compatible stealth coating coverage on critical areas of combat equipment, such as the leading and trailing edges of wings, fuselage edges, air intakes, cockpits, and engine tail nozzles, to reduce the likelihood of target detection by the enemy (Fig. 6b). In the realm of IR detection signals, the MXene layer prevents the thermal radiation signals generated by the target substrate from entering the environment and thus avoiding detection. The conductivity of the MXene layer is crucial in weakening the IR radiation characteristics and concealing high-temperature targets. Additionally, the wrinkled structure creates a diffuse reflection effect, scattering incoming IR waves in all directions, thereby reducing the likelihood of IR radiation signals reaching the target. In the scenario of radar detection signals, impedance matching is essential for the energy loss of EMWs. The wrinkled MCF is designed to match impedance with the input impedance effectively to optimize energy transmission. Through interactions with radar signals, the strong magnetic coupling and magnetic resonance phenomena of Fe_3_O_4_@C continuously dissipate EMW energy [57, 58]. Simultaneously, the interfacial polarization effect formed by the core–shell structure also contributes to attenuating the EMW energy [59, 60]. The differently shaped Fe_3_O_4_@C nanoparticles distributed in PDMS form a conductive network, leading to conduction losses [61, 62]. Therefore, applying the wrinkled MCF to the target surface allows for resistance against both radar and IR detection.

Considering the actual application environment, the adhesion of external substances such as dust, dirt, and water droplets can affect the device’s ability to reflect or absorb EMWs. Therefore, the self-cleaning capability achieved through the automatic removal of adherent substances ensures that stealth devices maintain highly efficient stealth performance over extended periods of use. Fortunately, the surface of MCF depicts excellent hydrophobicity, with a water contact angle of approximately 129° (Fig. 6c). This hydrophobic characteristic ensures normal operation even in humid environments, possessing self-cleaning capabilities. The wrinkled MCF remains undamaged, highlighting its remarkable flexibility and stability even after unfolding and uncurling in water. In certain curved surface applications within stealth technology, the aircraft fuselage and wing related components involved often have complex curvatures and irregular shapes. As a result, stealth devices require a degree of flexibility to conform to these surfaces. The designed MCF is capable of adapting to changes in finger curvature, providing seamless coverage over the target substrate (Fig. 6d). Furthermore, stealth devices need to withstand factors such as high-speed airflow impacts and external collisions during flight to achieve enduring stealth effectiveness. The film exhibits outstanding abrasion resistance when encountering external impact, swiftly returning to its primordial state (≈ 5 s, Fig. 6d and Video S4).

## Conclusions

In summary, we introduce a conception for multiscale hierarchical structure design aimed at achieving radar-IR compatible stealth. This camouflage strategy relies on the interface polarization and magneto-electric synergistic effect of Fe_3_O_4_@C in the radar absorbing layer, as well as the high reflectivity and low emissivity of microscale wrinkled MXene in the IR shielding layer. The diffuse reflection effect of wrinkled MXene layers on IR waves and their high transparency to MWs, along with a thermal insulation effect, have been verified through both theoretical simulations and experimental tests. By optimizing the morphology anisotropy, lattice structure, and EM parameters of Fe_3_O_4_@C/PDMS, the research has successfully achieved low-frequency MWA (< 8 GHz) and effective coverage in the X-band (8–12 GHz). The hierarchical wrinkled MCF demonstrates reduced IR emissivity to 0.35, thermal insulation capacity with a temperature difference (ΔT) of 70 °C, and a maximum RCS reduction of up to  − 20 dB m^2^. Furthermore, the MCF displays excellent flexibility, hydrophobicity (contact angle ≈ 129°), and abrasion resistance (recovery time ≈ 5 s), making it an ideal radar-IR compatible stealth candidate for long-term military and weapon applications. Multiscale hierarchical structure design for compatible stealth will become one of the research hotspots in modern complex combat environments.

## Supplementary Information

Below is the link to the electronic supplementary material.

Supplementary file1 (DOCX 5675 KB)

Supplementary file2 (MP4 4702 KB)

Supplementary file3 (MP4 705 KB)

Supplementary file4 (MP4 2380 KB)

Supplementary file5 (MP4 1928 KB)
