# Supplementary material for: Magneto-Dielectric Synergy and Multiscale Hierarchical Structure Design Enable Flexible Multipurpose Microwave Absorption and Infrared Stealth Compatibility
Source: Nanomicro Lett. 2024 Oct 16;17:40. doi: 10.1007/s40820-024-01549-4 (PMC11480309; doi:10.1007/s40820-024-01549-4)
Supplement: Supplementary file 1 — Supplementary file1 (DOCX 5675 KB) [file 40820_2024_1549_MOESM1_ESM.docx]

Supporting Information for

**Magneto-Dielectric Synergy and** **Multiscale Hierarchical Structure Design Enable Flexible Multipurpose Microwave Absorption and Infrared Stealth Compatibility**

Chen Li^1^, Leilei Liang^1^, Baoshan Zhang^1,^*, Yi Yang^1,^*, Guangbin Ji^2,^*

^1^School of Electronic Science and Engineering, Nanjing University, Nanjing 210093, P. R. China

^2^College of Materials Science and Technology, Nanjing University of Aeronautics and Astronautics, Nanjing 210016, P. R. China

*Corresponding authors. E-mail: [bszhang@nju.edu.cn](mailto:bszhang@nju.edu.cn) (Baoshan Zhang); [malab@nju.edu.cn](mailto:malab@nju.edu.cn) (Yi Yang); [gbji@nuaa.edu.cn](mailto:gbji@nuaa.edu.cn) (Guangbin Ji)

**Note S1 Theoretical Analyses of Relevant Parameters**

Strain effects are described by the Gibbs-Thomson equation:

$\text{u}\left(R\right)-\text{u}\left(\infty\right)=2\gamma\Omega/R$ (S-1)

Where $\text{u}\left(R\right)$ and $\text{u}\left(\infty\right)$ respectively represent the molar free energy and volume of particles with a radius of *R*, $\gamma$ and $\Omega$ denote the surface free energy and volume of atoms.

The reflection loss (*RL*) can be expressed by the following equation:

$RL=20\log\left|\frac{Z_{in}-Z_{0}}{Z_{in}+Z_{0}}\right|$ (S-2)

$Z_{in}=Z_{0}\sqrt{\frac{\mu}{\varepsilon}}\tanh\left(j\frac{2\pi fd\sqrt{\mu\varepsilon}}{c}\right)$ (S-3)

where *Z_in_* and *Z_0_* are impedance of input impedance and free space of the absorber, *f* is the frequency of the incident wave, $\mu$ is the permeability of the material, $\varepsilon$ is the permittivity of the material, dm is the thickness of the material, and *c* is the speed of light.

The dielectric loss tangent and magnetic loss tangent can be expressed as:

$\tan\delta_{\varepsilon}=\varepsilon"/\varepsilon'$ (S-4)

$\tan\delta_{\mu}=\mu"/\mu'$ (S-5)

Eddy current losses can be expressed as:

$C_{0}=\mu"\left(\mu'\right)^{-2}f^{-1}$ (S-6)

When the value of *C_0_* remains constant regardless of frequency, it indicates that magnetic losses are solely caused by eddy current effects.

Polarization effect can be evaluated using the Debye equation:

$\left(\varepsilon'-\frac{\varepsilon_{s}+\varepsilon_{\infty}}{2}\right)^{2}+\left(\varepsilon''\right)^{2}=\left(\frac{\varepsilon_{s}-\varepsilon_{\infty}}{2}\right)^{2}$ (S-7)

where $\varepsilon_{\infty}$ and $\varepsilon_{s}$ are the relative dielectric permittivity at the high frequency limit and static permittivity. The semicircle obtained from the above equation is defined as a Cole-Cole semicircle, where each semicircle corresponds to a Debye relaxation process.

Attenuation constant can be expressed as:

$\alpha=\frac{\sqrt{2}\pi f}{c}\sqrt{\left(\mu''\varepsilon''-\mu'\varepsilon'\right)+\sqrt{\left(\mu''\varepsilon''-\mu'\varepsilon'\right)^{2}+\left(\varepsilon'\mu''+\varepsilon''\mu'\right)^{2}}}$ (S-8)

Impedance matching (*Z*) can be expressed as:

$Z=\left|\frac{Z_{in}}{Z_{0}}\right|$ (S-9)

According to transmission line theory, if the characteristic impedance of the transmission line does not match the load impedance, reflections will occur at the load end. In essence, the degree of impedance matching determines whether EMWs can smoothly enter the absorber material. As the *Z* approaches 1, the impedance matching improves.

Radar cross-section (RCS) can be expressed as:

$\sigma\left(dB\cdot m^{2}\right)=10\lg\left(4\pi\lim_{R\to\infty} R^{2}\frac{\left|E^{s}\right|^{2}}{\left|E^{i}\right|^{2}}\right)$ (S-10)

Where $E^{s}$ and $E^{i}$ represent the intensities of the scattered electric field and incident electric field, respectively, and *R* is the detection distance.

The energy detected by the IR camera can be expressed as:

$P_{\det\text{ection}}=\varepsilon_{\theta}P_{BB_{-}\theta}\left(T_{\text{t}\arg\text{et}}\right)+R_{\theta}P_{\text{source}}$ (S-11)

Where $\varepsilon_{\theta}$ and $R_{\theta}$ represent the directional emissivity and directional reflectivity of the device, $P_{BB_{-}\theta}$ and $P_{\text{source}}$ are the radiation intensities of the black body and the external heat source, and $T_{\text{t}\arg\text{et}}$ denotes the target temperature.

From infrared physics, it is known that the infrared radiant energy of an object conforms to the Stefan-Boltzmann law:

$W=\varepsilon\sigma T^{4}$ (S-12)

The total radiative energy of an object is denoted as *W*, where $\sigma$ represents the Boltzmann constant, $\varepsilon$ signifies the IR emissivity of object, and *T* stands for the absolute temperature of the object.

**Supplementary Tables**

**Table S1** Elemental content distribution of Fe_3_O_4_@C

| **Element** | **Atomic Fraction (%)** | **Atomic Error (%)** | **Mass Fraction (%)** | **Mass Error (%)** |
| --- | --- | --- | --- | --- |
| C | 24.41 | 4.24 | 11.11 | 1.12 |
| O | 47.08 | 12.44 | 28.54 | 6.37 |
| Fe | 28.51 | 6.21 | 60.34 | 9.99 |

**Table S2** Thermal conductivity test report of Fe_3_O_4_@C/PDMS film

| ***T* (℃)** | $\boldsymbol{\gamma}$ **(g/cm^3^)** | ***c* (kj/kg*k)** | $\boldsymbol{\Lambda}$ **(mm^2^/S)** | ***S* (W/m^2^k)** | ***K* (W/m•K)** |
| --- | --- | --- | --- | --- | --- |
| 100.3 | 0.85 | 0.889 | 0.2942 | 4.427 | 0.2825 |

**Table S3** Comparative plots of the radar-IR compatible stealth performance of previously reported and our works

| **Names of Sample** | **RL (dB)** | **EAB (GHz)** | **Emissivity** | **Refs.** |
| --- | --- | --- | --- | --- |
| Cr_2_O_3_@ATO | -14.50 | 2.00 | 0.85 | [S1] |
| CuS/ZnS-GO | -26.00 | 6.38 | 0.58 | [S2] |
| CIP/PU | -32.00 | 6.84 | 0.64 | [S3] |
| PRF | -23.00 | 1.50 | 0.39 | [S4] |
| VO_2_/CA | -52.00 | 5.20 | 0.58 | [S5] |
| ITO | -20.07 | 3.83 | 0.64 | [S6] |
| Ni/SiC NWs | -49.29 | 4.85 | 0.89 | [S7] |
| CoFe_2_O_4_/SiO_2_/ZAO | -59.31 | 4.00 | 0.71 | [S8] |
| CuS@MoS_2_/C | -48.40 | 6.62 | 0.54 | [S9] |
| rGO/CuS@SA | -52.40 | 5.75 | 0.59 | [S10] |
| Fe_3_O_4_@SnO_2_ | -39.40 | 1.70 | 0.37 | [S11] |
| HMCS@f-Fe_2_O_3_ | -34.16 | 4.80 | 0.51 | [S12] |
| S4 | -58.28 | 4.93 | 0.38 | This work |
| S5 | -61.47 | 4.93 | 0.45 | This work |
| S6 | -64.92 | 4.04 | 0.35 | This work |

**Table S4** Comparative plots of radar scattering cross section and IR thermal camouflage capability of previously reported and our works

| **Names of Sample** | **RCS_decrease_(dB·m²)** | **ΔT (℃)** | **Refs.** |
| --- | --- | --- | --- |
| Shaddock peel-based aerogel | 16.28 | 34.00 | [S16] |
| Carbon SiO_2_@CNTs aerogel | 26.18 | 51.00 | [S17] |
| 3D porous carbon aerogel | 15.86 | 45.50 | [S18] |
| CBC/Cu/TiO_2_ aerogel | 23.88 | 43.00 | [S19] |
| Co/N-doped C@carbon | 15.25 | 55.00 | [S20] |
| MXene/C aerogels | 12.02 | 39.00 | [S21] |
| Multifrequency camouflage system | 20.00 | 10.00 | [S22] |
| Graphene/polyimide/Co-N-C | 10.00 | 80.00 | [S23] |
| S4 | 20.02 | 62.80 | This work |
| S5 | 20.78 | 63.70 | This work |
| S6 | 11.08 | 69.60 | This work |

**Supplementary Figures**


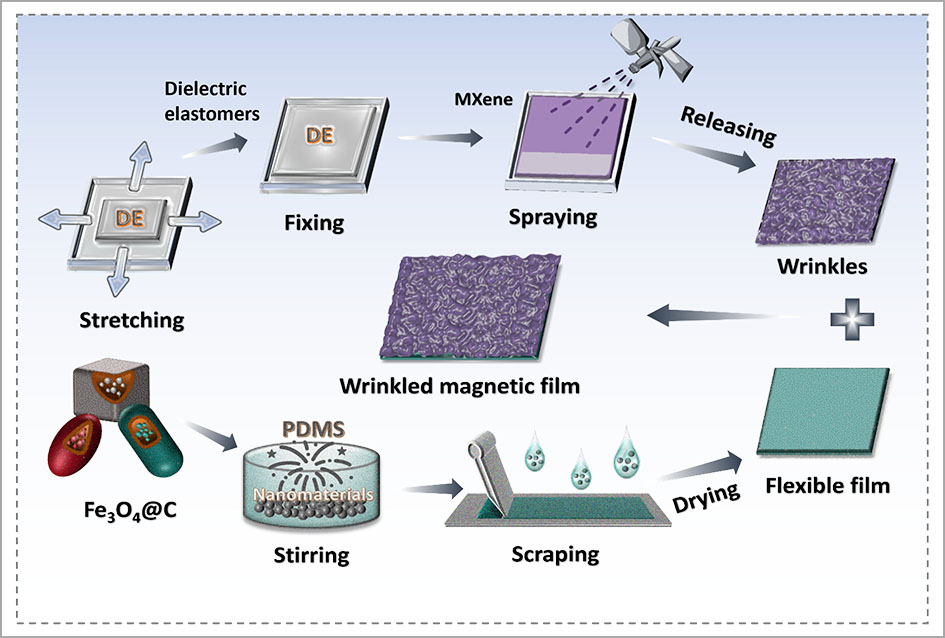


**Fig. S1** Fabricated schemes of multiscale hierarchical wrinkled magnetic composite films (MCFs)


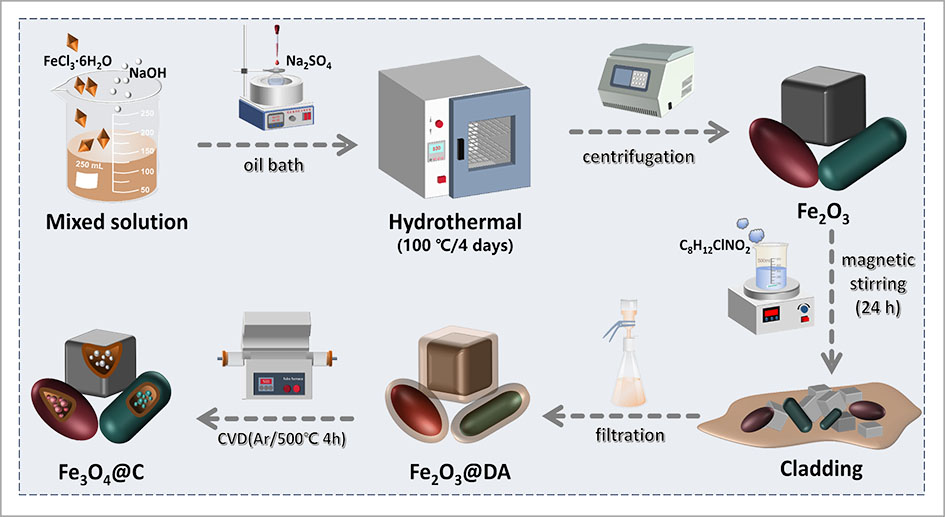


**Fig. S2** Fabricated schemes of Fe_3_O_4_@C NPs

The preparation process of Fe_3_O_4_@C is illustrated in Fig. S2, where diverse morphologies of Fe_2_O_3_ precursors (cubic, ellipsoidal, and peanut-shaped) are obtained by varying the concentration of sodium sulfate (Na_2_SO_4_) during oil bath treatment. Dopamine hydrochloride is then added and magnetically stirred to envelop an organic layer, followed by high-temperature annealing to yield Fe_3_O_4_@C.


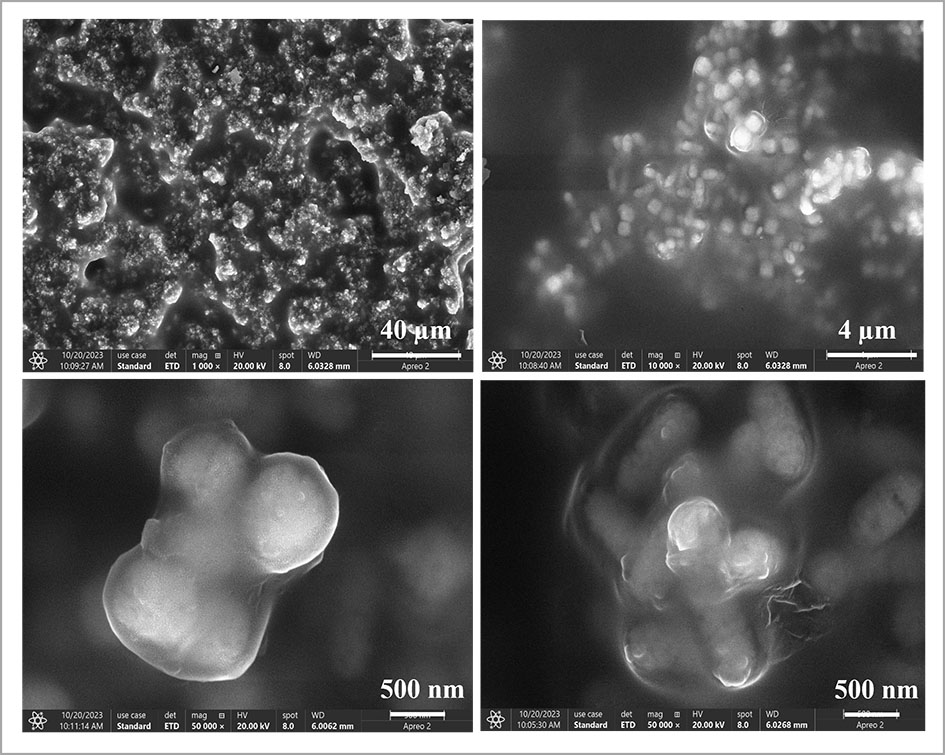


**Fig. S3** Typical SEM images of Fe_3_O_4_@C/PDMS films


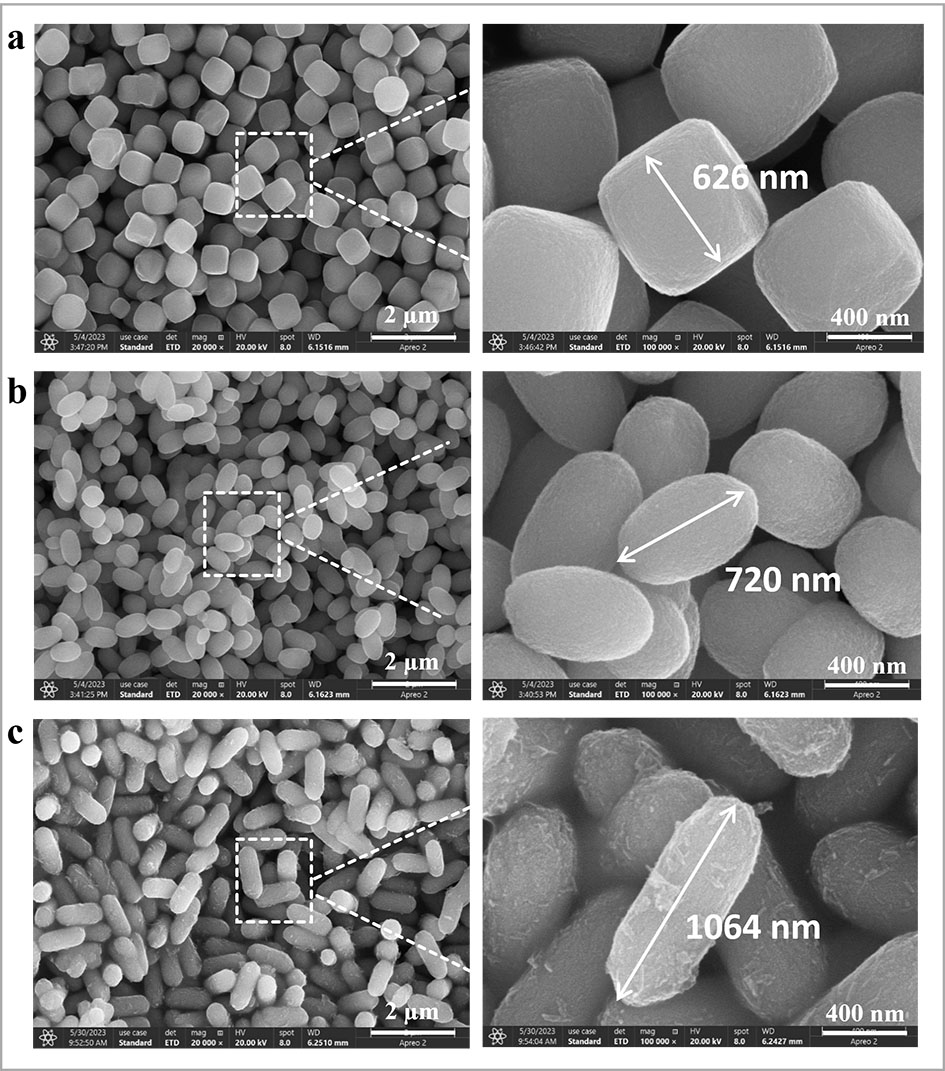


**Fig. S4** Typical SEM images of Fe_2_O_3_. **a** cubic (0.0 M), **b** ellipsoidal (0.6 M), and **c** peanut-shaped (1.2 M)

Fe_3_O_4_@C NPs were synthesized from Fe_2_O_3_ precursor, with their morphology altering from cubic to ellipsoidal and peanut-shaped as the Na_2_SO_4_ concentration increased. The uniform size and smooth surfaces of the synthesized NPs were evident in Fig. S4a-c. The diameter of the cubic is approximately 626 nm, while the long diameters of the ellipsoidal and peanut-shaped are about 720 and 1064 nm, respectively.


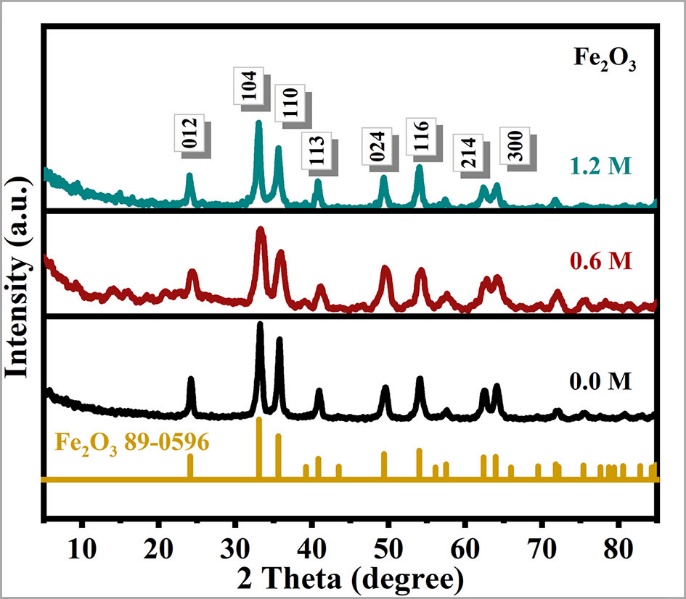


**Fig. S5** XRD pattern of cubic (0.0 M), ellipsoidal (0.6 M), and peanut-shaped (1.2 M) Fe_2_O_3_


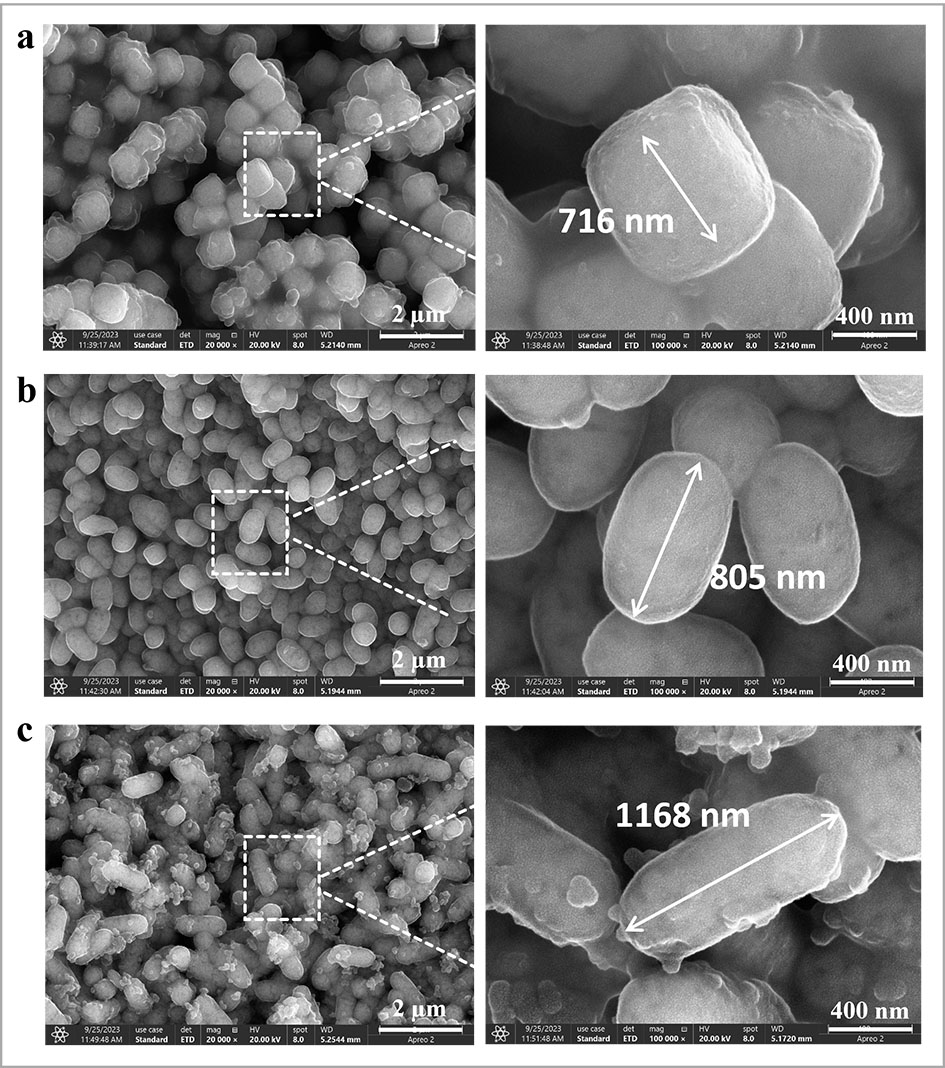


**Fig. S6** Typical SEM images of Fe_3_O_4_@C. **a** cubic (0.0 M), **b** ellipsoidal (0.6 M), and **c** peanut-shaped (1.2 M)

The carbon coating applied on the precursor resulted in a smoother surface, as demonstrated in the SEM images in Fig. S6. The diameter of the cubic is approximately 716 nm, while the long diameters of the ellipsoidal and peanut-shaped are about 805 and 1168 nm, respectively.


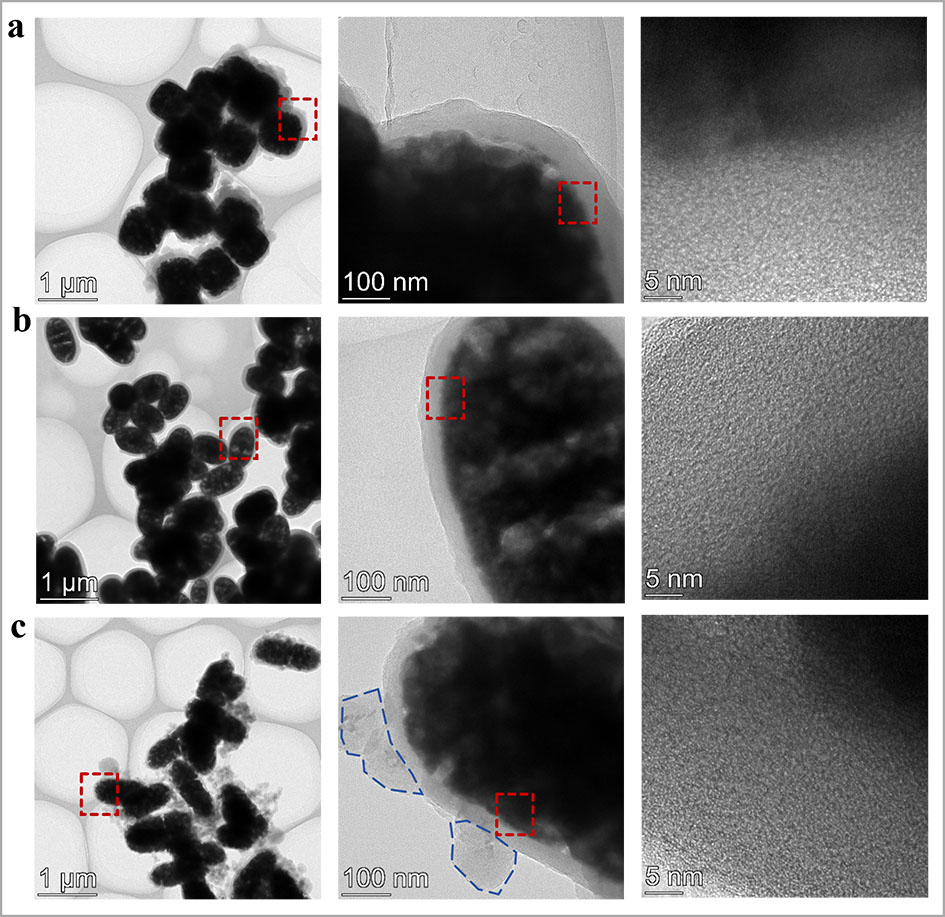


**Fig. S7** Typical TEM images of Fe_3_O_4_@C. **a** cubic (0.0 M), **b** ellipsoidal (0.6 M), and **c** peanut-shaped (1.2 M)


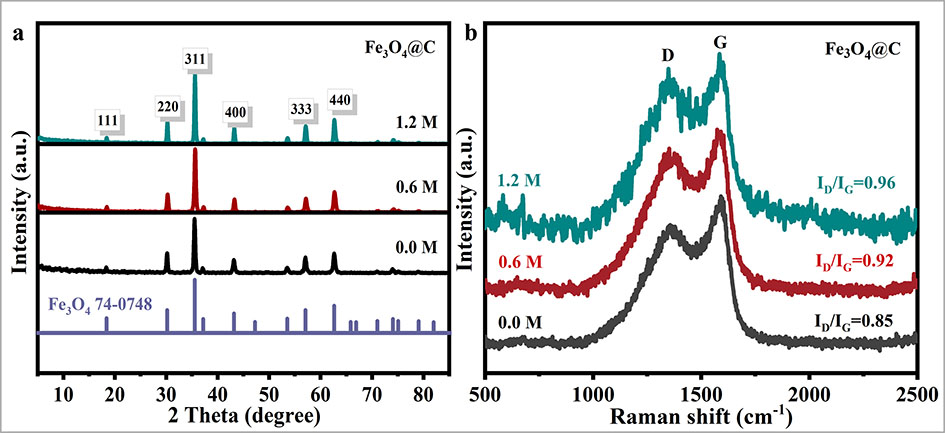


**Fig. S8 a** XRD pattern and **b** Raman spectrogram of cubic (0.0 M), ellipsoidal (0.6 M), and peanut-shaped (1.2 M) Fe_3_O_4_@C

XRD images show characteristic peaks at 18.29°, 30.09°, 35.44°, 43.07°, 56.96°, and 62.54°, corresponding to the (111), (220), (311), (400), (333), and (440) crystal planes of Fe_3_O_4_ (74-0748), confirming the presence of Fe_3_O_4_ (Fig. S8a). However, the diffraction peaks of carbon cannot be manifested in XRD because of the amorphous carbon layer [S13].


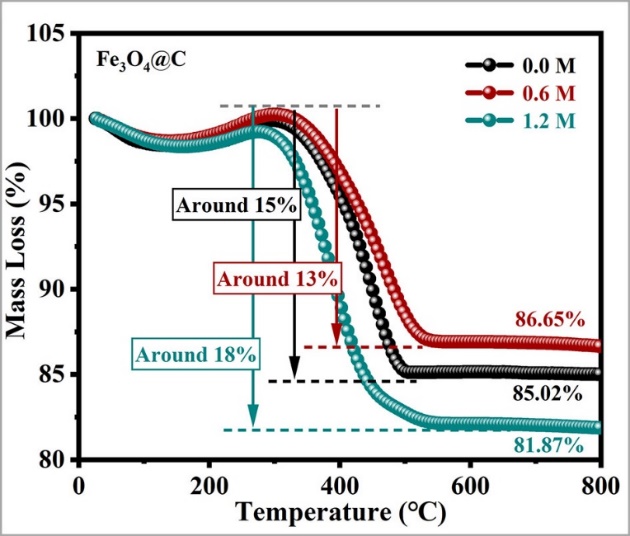


**Fig. S9** TG curves of cubic (0.0 M), ellipsoidal (0.6 M), and peanut-shaped (1.2 M) Fe_3_O_4_@C

The thermogravimetric analysis (TGA) of Fe_3_O_4_@C within the temperature range of 0-800 °C demonstrates a slight decrease in weight loss below 100 °C, attributed to the removal of adsorbed water within the material (Fig. S9). A subtle weight increase in the temperature range of 100-300 °C originates from the oxidation of Fe_3_O_4_. The substantial weight loss between 300 and 500 °C mainly results from the combustion of the carbon shell. Notably, the peanut-shaped NPs exhibits higher weight loss of approximately 18 wt%, indicating higher carbon content.


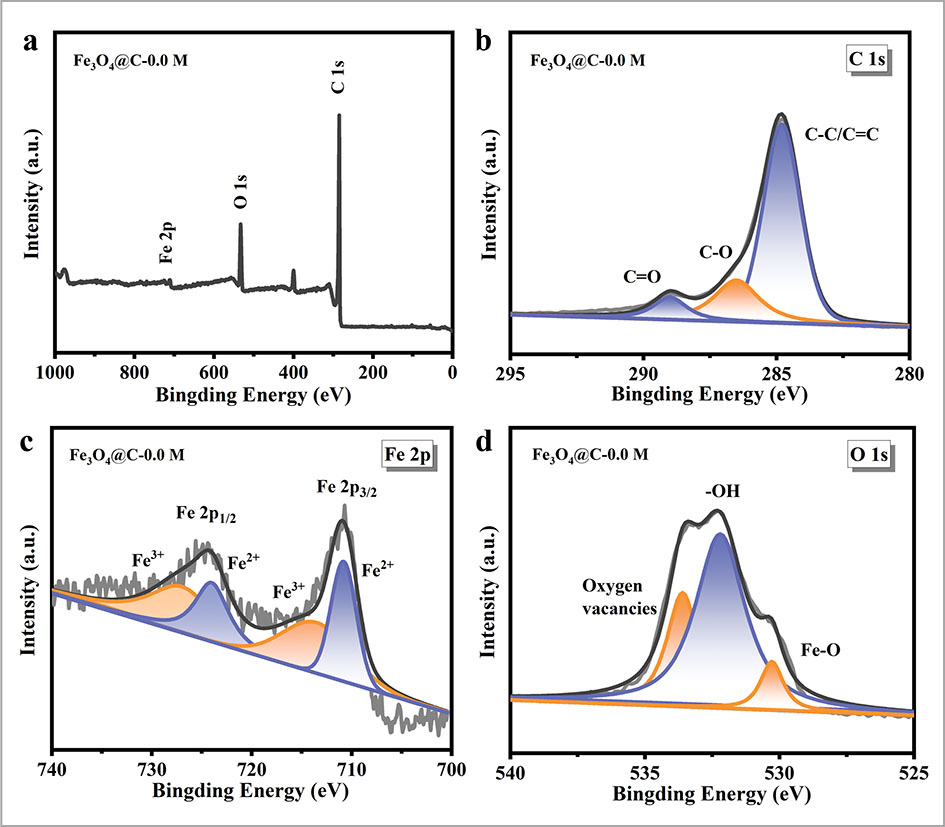


**Fig. S10 a-d** High resolution spectra of C 1s, Fe 2p and O 1s for cubic (0.0 M) Fe_3_O_4_@C


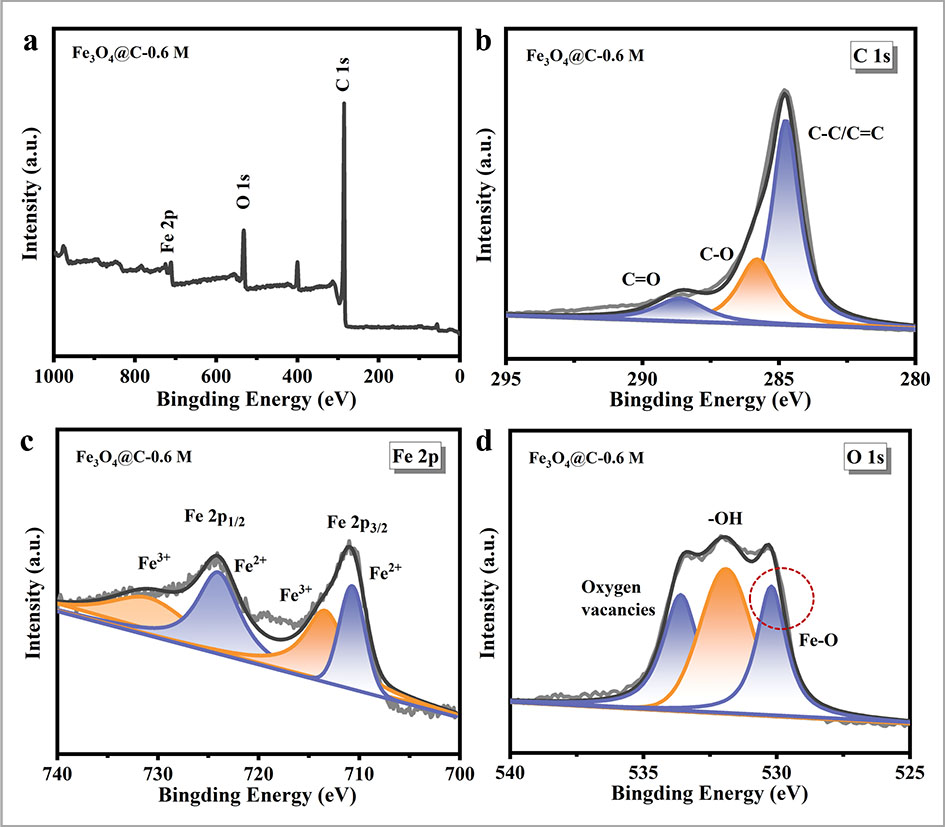


**Fig. S11 a-d** High resolution spectra of C 1s, Fe 2p and O 1s for ellipsoidal (0.6 M) Fe_3_O_4_@C


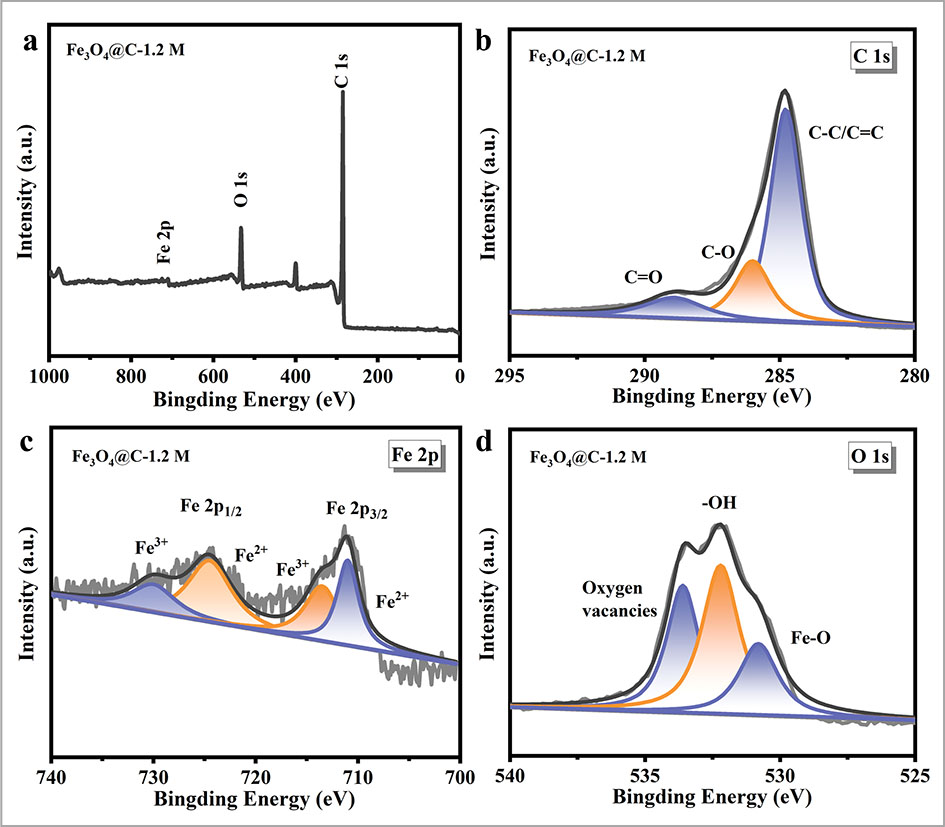


**Fig. S12 a-d** High resolution spectra of C 1s, Fe 2p and O 1s for peanut-shaped (1.2 M) Fe_3_O_4_@C

The XPS survey (Fig. S10a) indicates the presence of C, Fe, and O atoms on the surface of the 0.0 M sample. In the high-resolution C 1s spectrum (Fig. S10b), the three peaks at 284.6, 286.3, and 288.7 eV are attributed to the C-C/C=C, C-O, and C=O groups, respectively [S14]. The high-resolution Fe 2p spectrum (Fig. S10c) shows the peaks at 711.8 and 713.9 eV corresponding to the binding energy of Fe 2p_3/2_ for Fe^2+^ and Fe^3+^, while the peaks at 724.1 and 727.4 eV belong to the binding energy of Fe 2p_1/2_ for Fe^2+^ and Fe^3+^ [S15]. The presence of Fe^2+^ and Fe^3+^ ions in Fe_3_O_4_ is confirmed. From Fig. S10d, O 1s peaks (530.2, 532.2, and 533.6 eV) correspond to lattice oxygen Fe-O, -OH, and oxygen vacancies, demonstrating the presence of oxygen-containing groups and iron oxide on the surface of Fe_3_O_4_@C [S13]. The existence of oxygen vacancies signifies the presence of oxygen vacancy defects in the nanomaterial, corroborating the results of Raman spectroscopy analysis. The XPS spectra of the prepared 0.6 M and 1.2 M Fe_3_O_4_@C samples (Figs. S11 and S12) are nearly identical to those of the 0.0 M sample, except for differences in the intensity of the Fe-O peak in O 1s. The intensity of the Fe-O peak exceeds that of the other two samples due to the structural integrity of the 0.6 M, as illustrated in Fig. S11d.


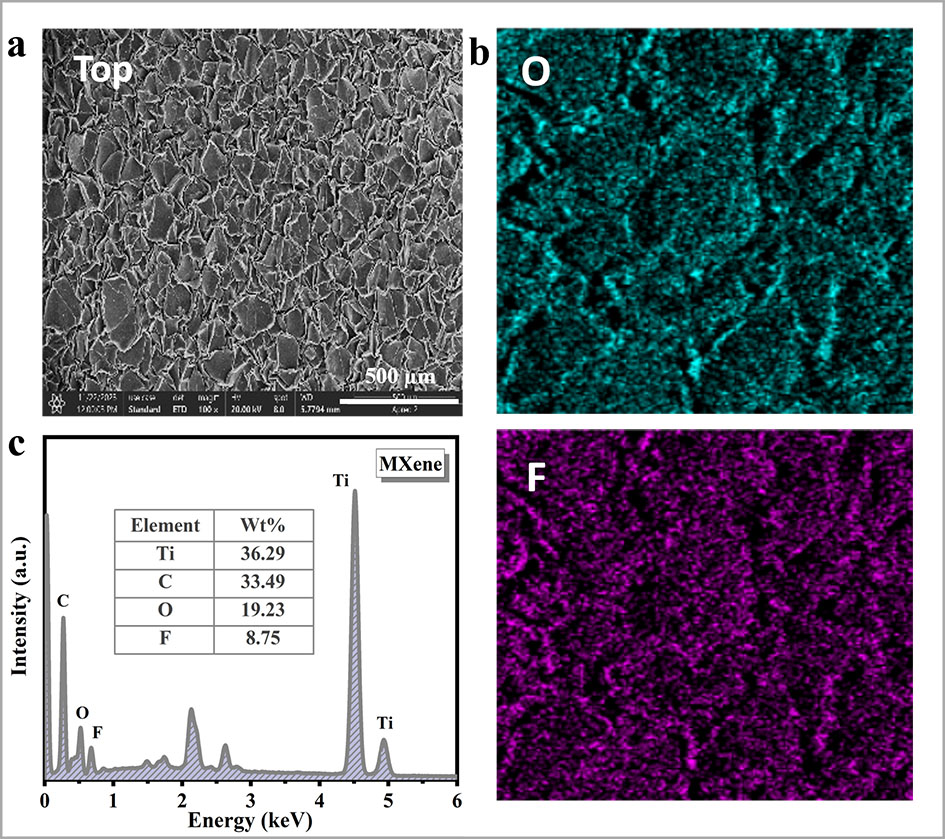


**Fig. S13 a-c** Typical SEM and EDS images of wrinkled MXene


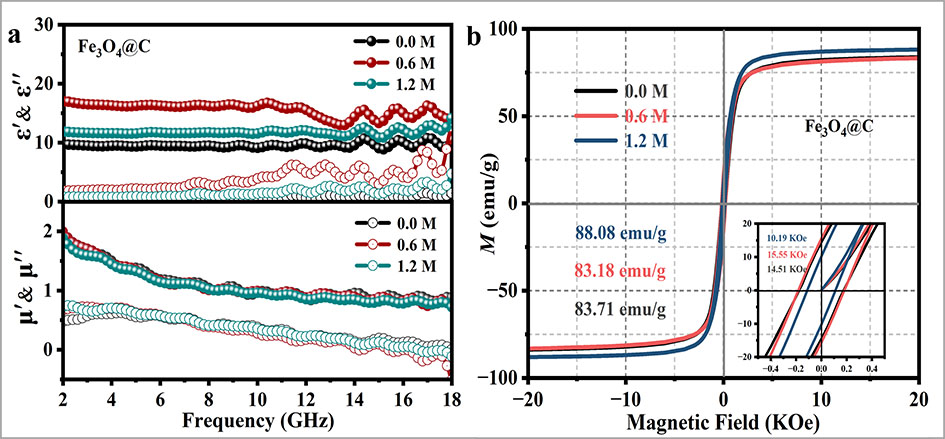


**Fig. S14 a** Complex permittivity and permeability, **b** VSM curves of Fe_3_O_4_@C (0.0 M, 0.6 M, and 1.2 M)


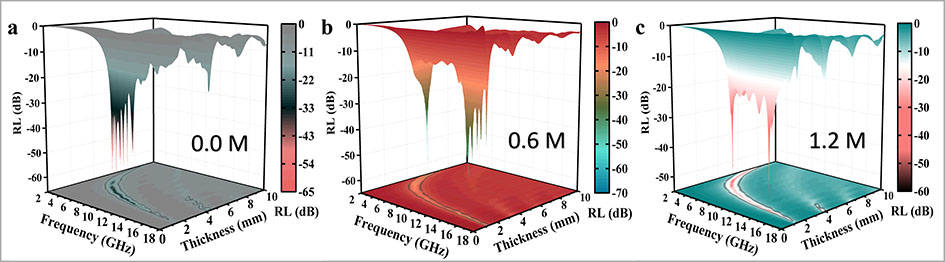


**Fig. S15** Three-dimensional color maps of Fe_3_O_4_@C. **a** cubic (0.0 M), **b** ellipsoidal (0.6 M), and **c** peanut-shaped (1.2 M)


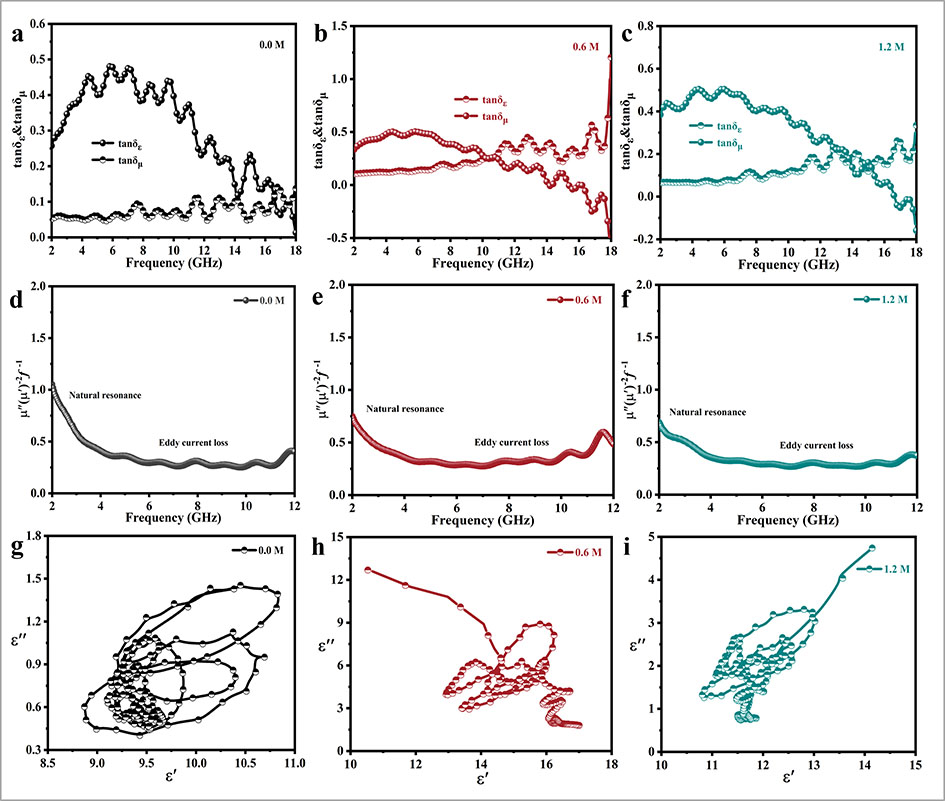


**Fig. S16 a-c** Dielectric and magnetic loss tangent, **d-f** Eddy current curve, and **g-i** Cole-Cole plots of Fe_3_O_4_@C


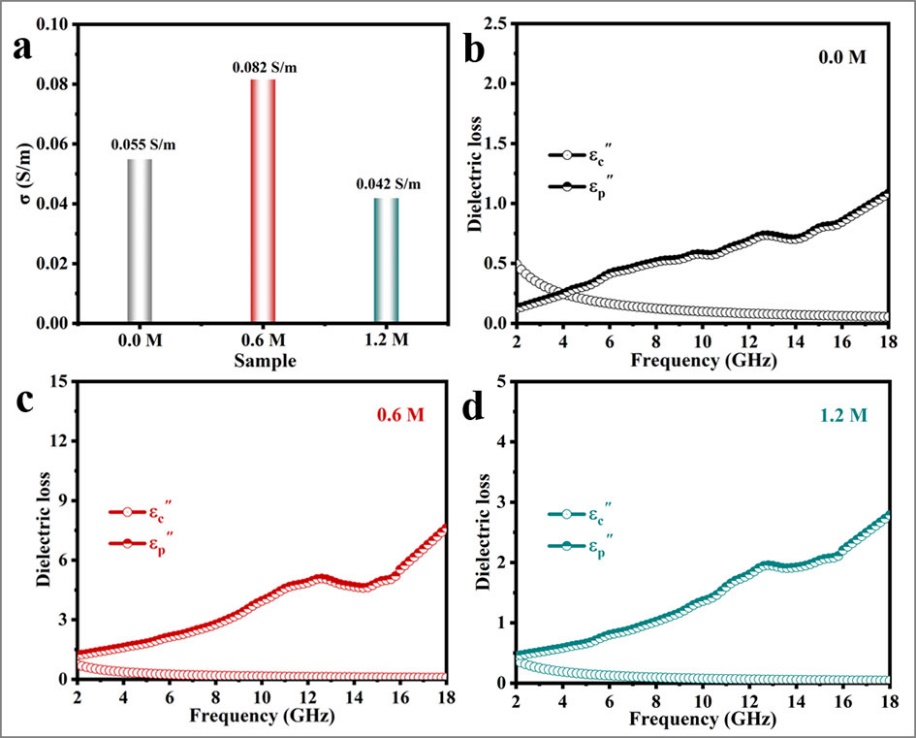


**Fig. S17** **a** Cconductivity, **b-d** $\varepsilon''_{c}\sim f$ and $\varepsilon''_{\text{p}}\sim f$ curves of Fe_3_O_4_@C


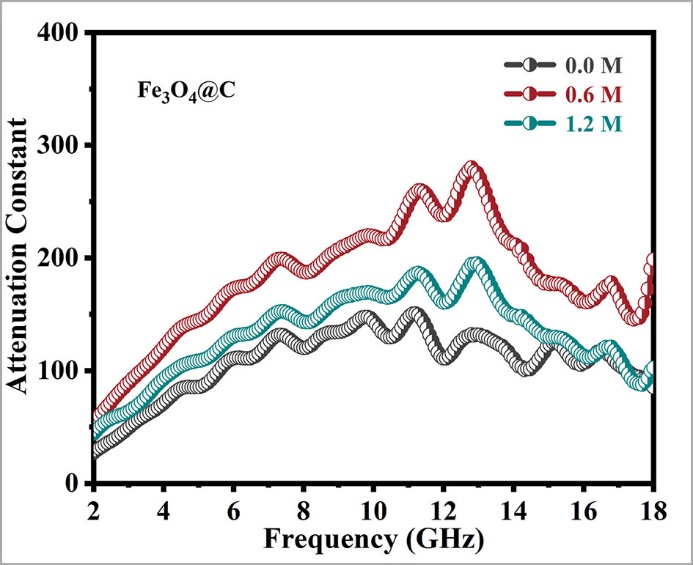


**Fig. S18** Attenuation constant of Fe_3_O_4_@C (0.0 M, 0.6 M, and 1.2 M)


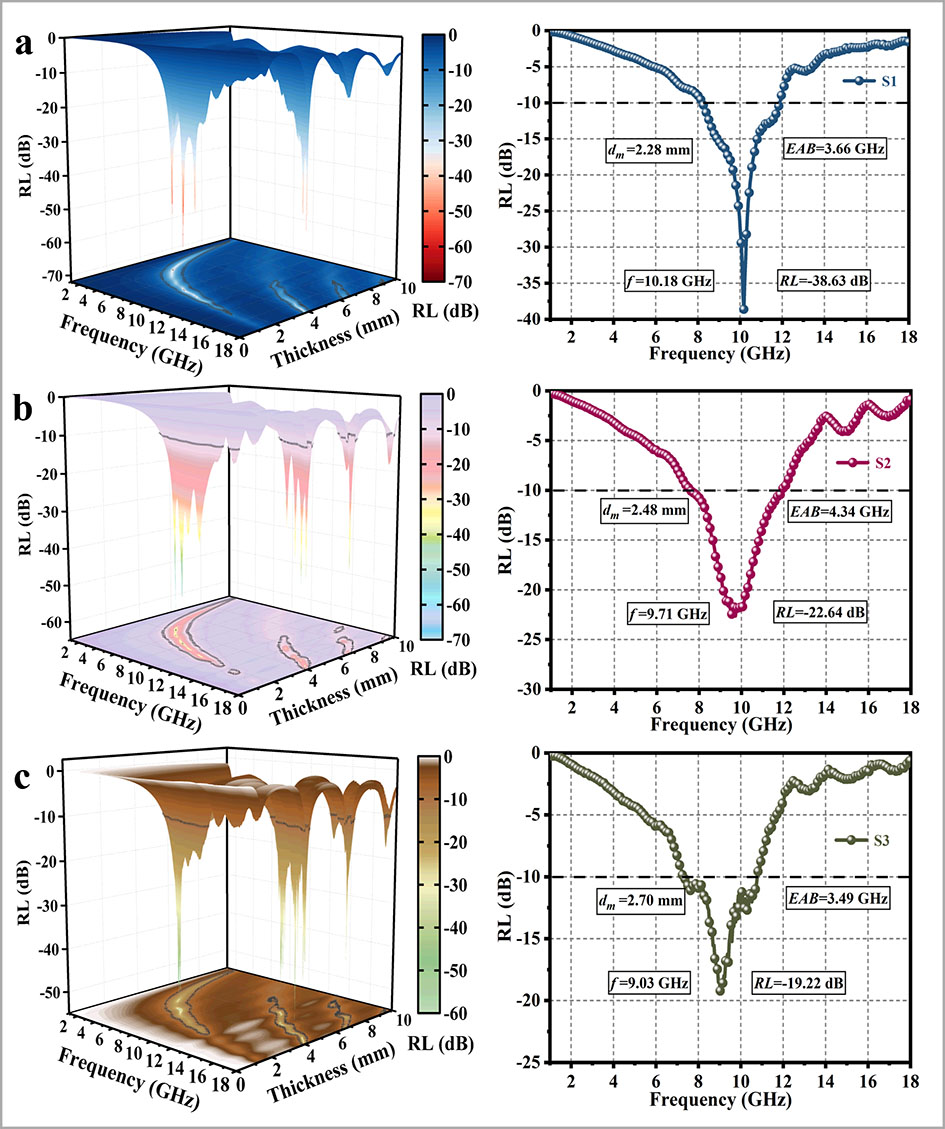


**Fig. S19** 3D color maps and typical 2D curves of **a** S1, **b** S2, and **c** S3


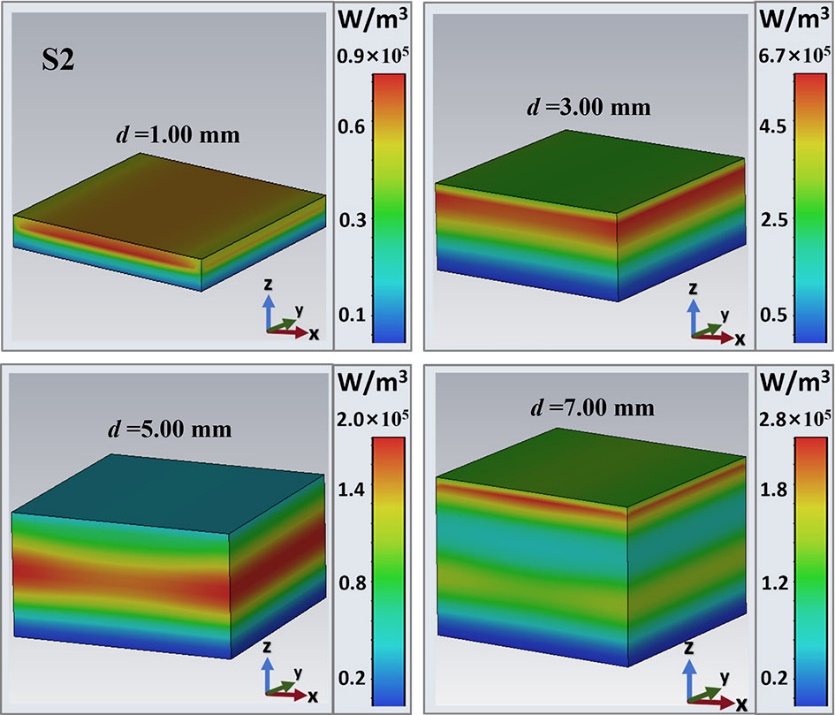


**Fig. S20** Electromagnetic power loss density map of S2 with different thickness derived from CST at 10 GHz


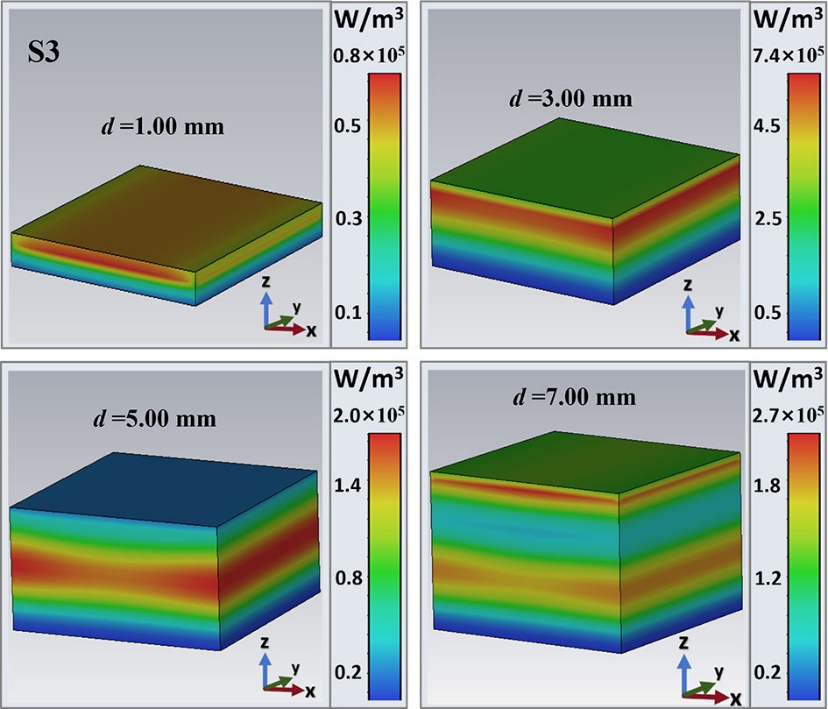


**Fig. S21** Electromagnetic power loss density map of S3 with different thickness derived from CST at 10 GHz


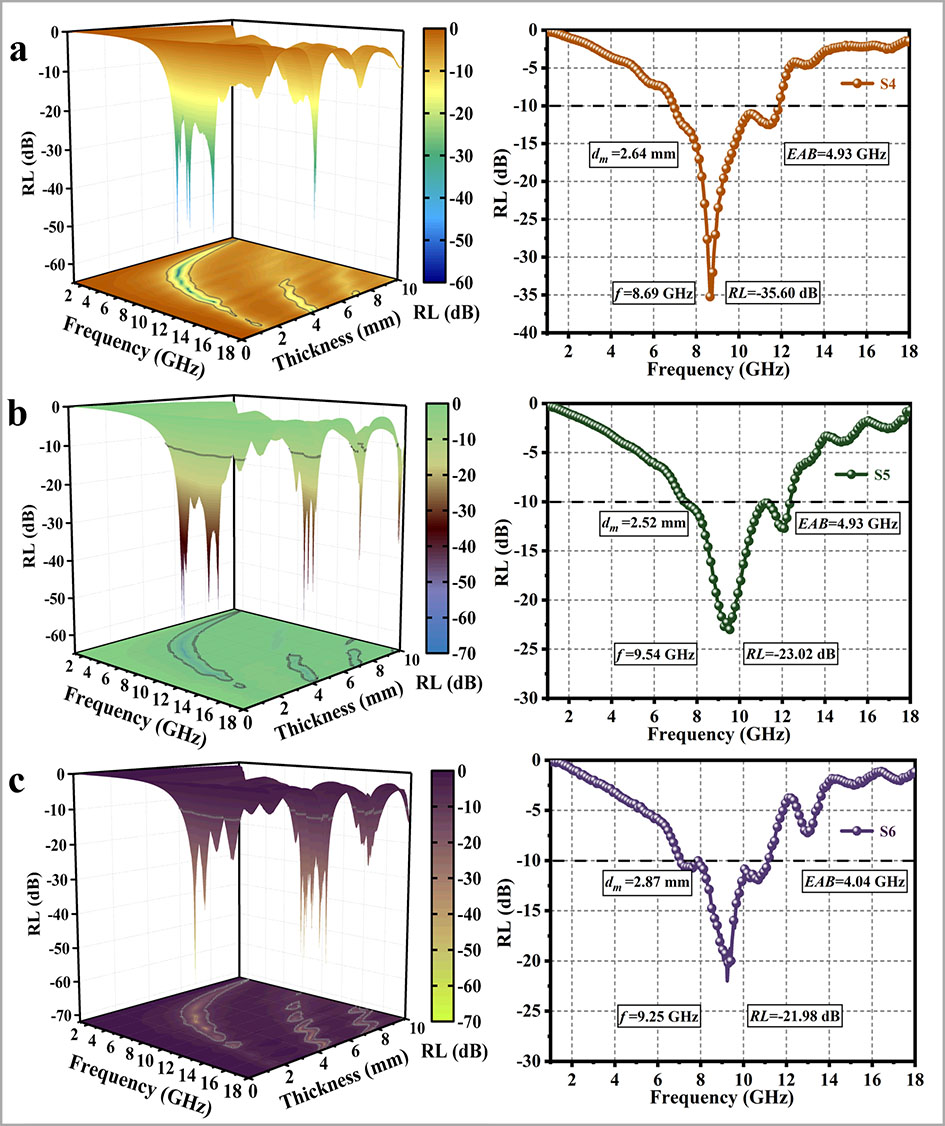


**Fig. S22** 3D color maps and typical 2D curves of **a** S4, **b** S5, and **c** S6


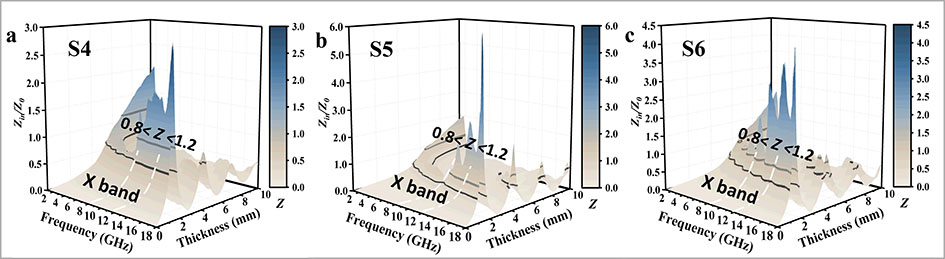


**Fig. S23** Impedance matching color maps of S4-S6


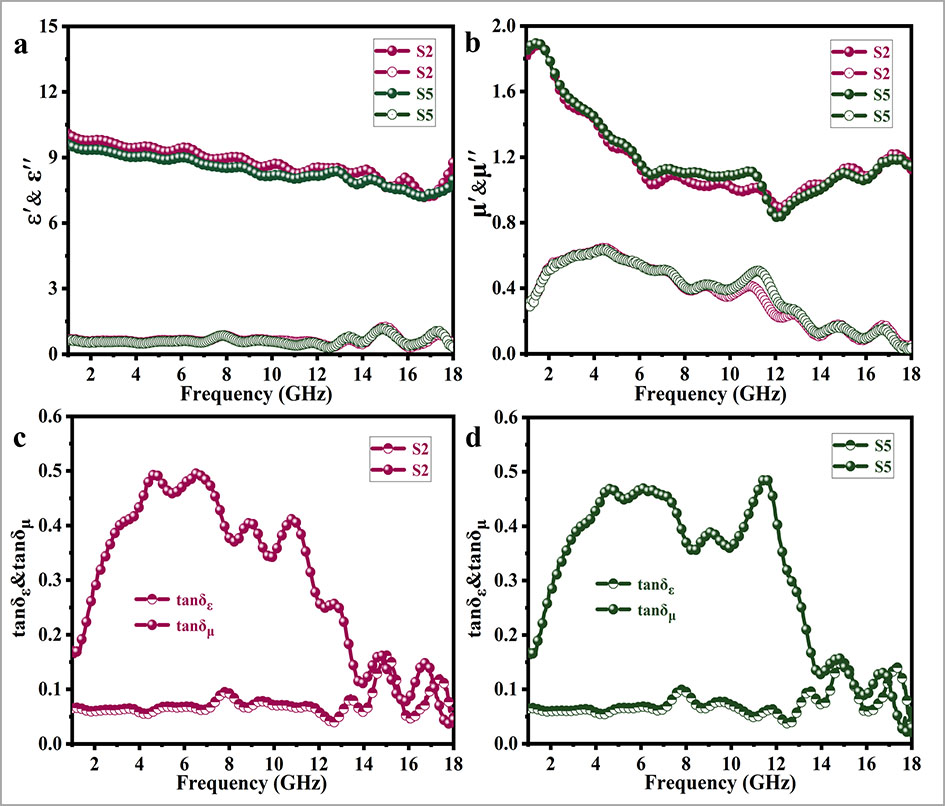


**Fig. S24 a** Complex permittivity, **b** Complex permeability, **c, d** Dielectric and magnetic loss tangent of S2 and S5


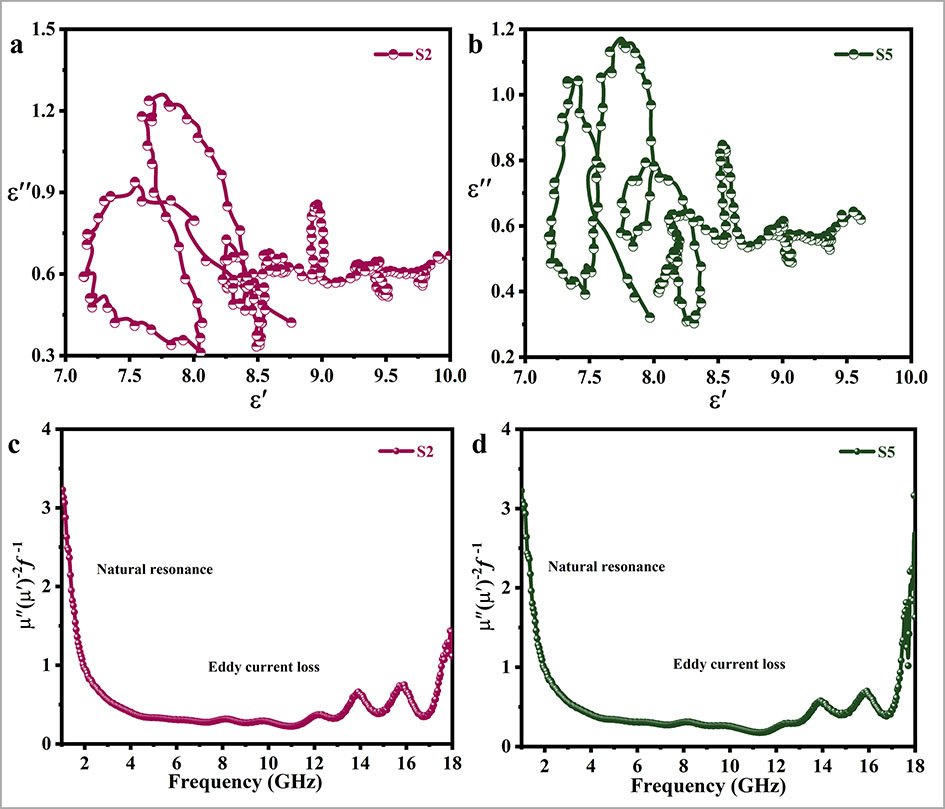


**Fig. S25 a, b** Cole-Cole plots and **c, d** Eddy current curve of S2 and S5


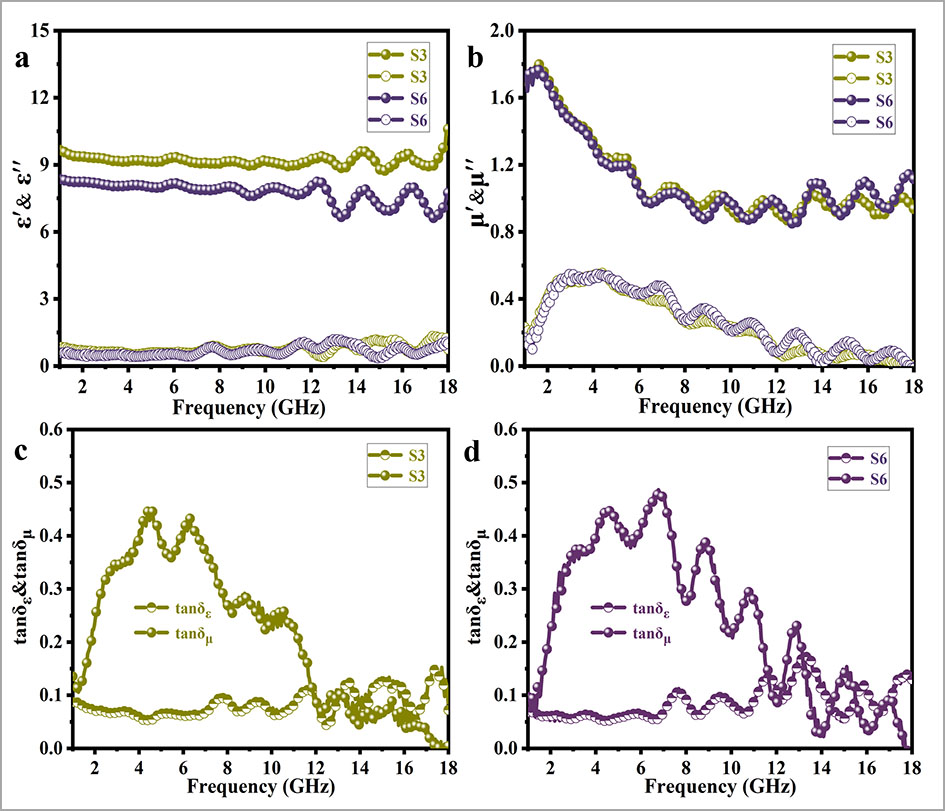


**Fig. S26 a** Complex permittivity, **b** Complex permeability, **c, d** Dielectric and magnetic loss tangent of S3 and S6


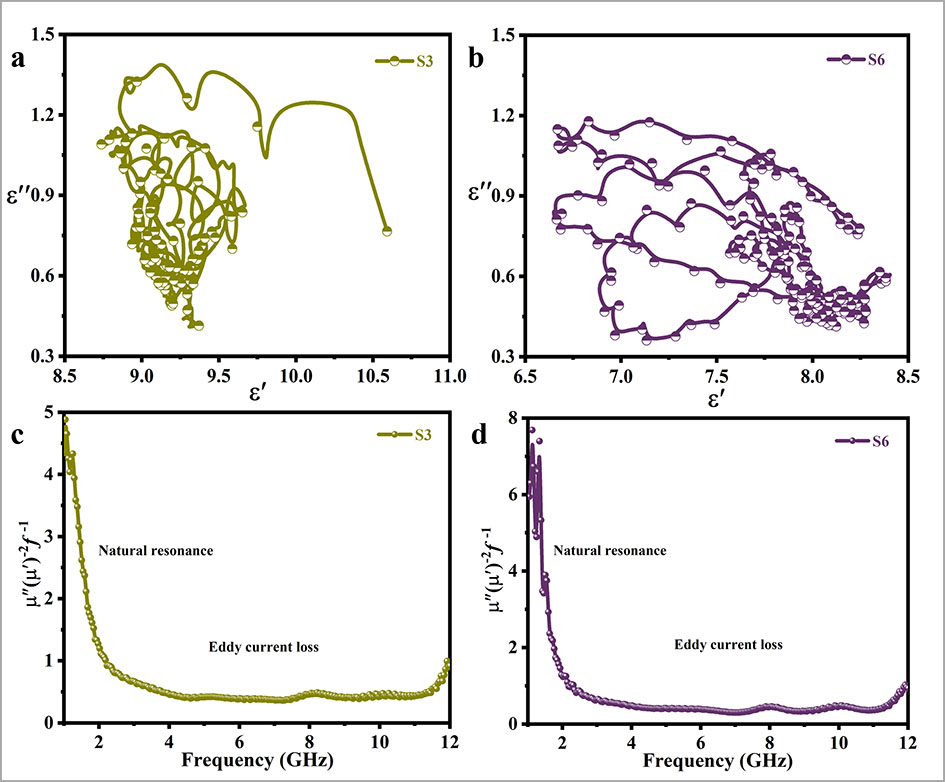


**Fig. S27 a, b** Cole-Cole plots and **c, d** Eddy current curve of S3 and S6


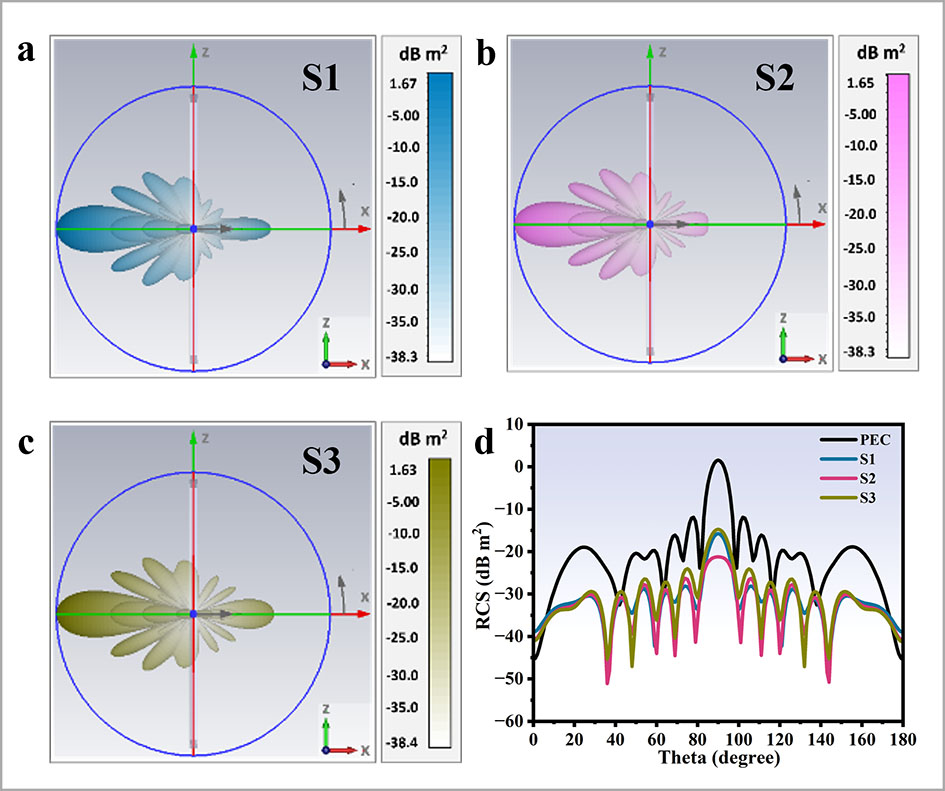


**Fig. S28** 3D radar wave scattering signals of **a** S1, **b** S2, and **c** S3. **d** RCS simulated curves of S1, S2, and S3


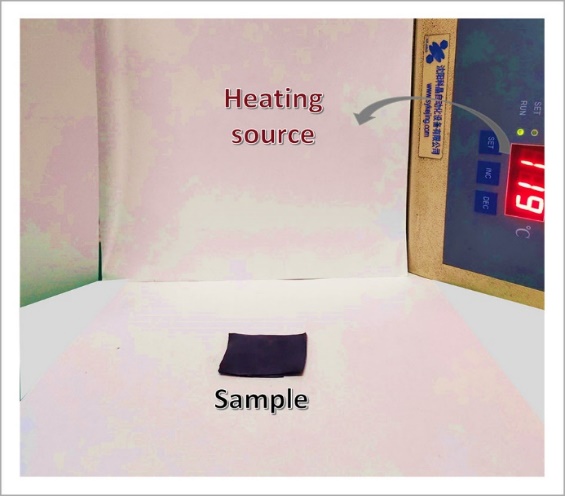


**Fig. S29** Digital photographs of the thermal IR images measurement setup under oblique incidence


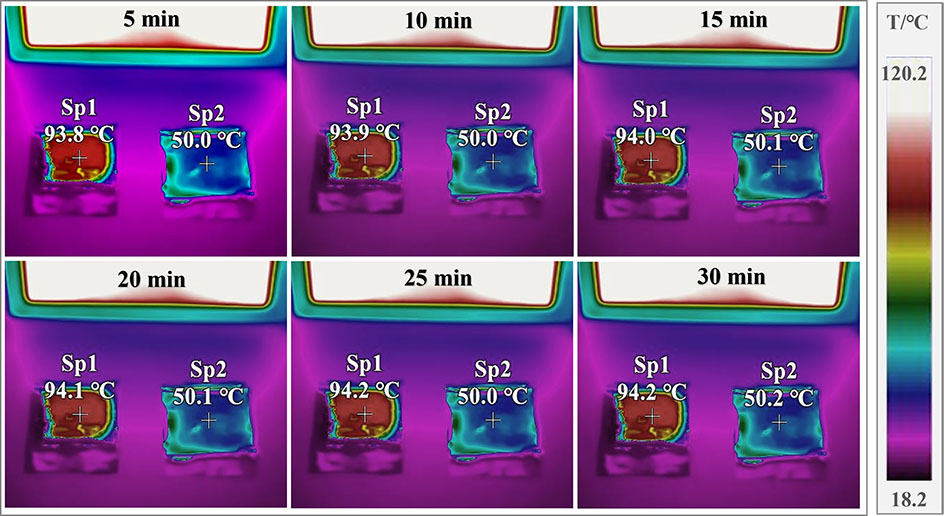


**Fig. S30** Thermal IR images of smooth (left) and wrinkled (right) MXene MCFs during 30 min


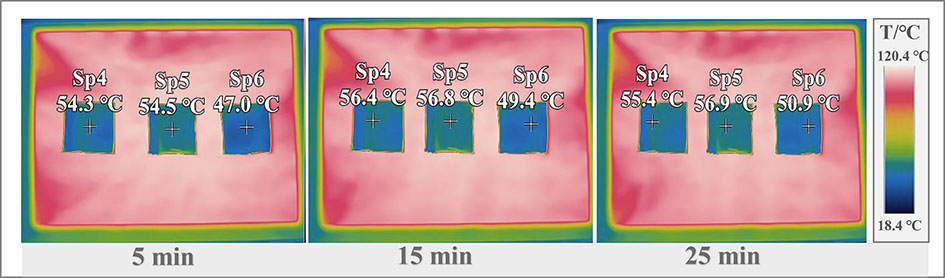


**Fig. S31** Thermal IR images of S4-S6 at 5 min, 15 min and 25 min


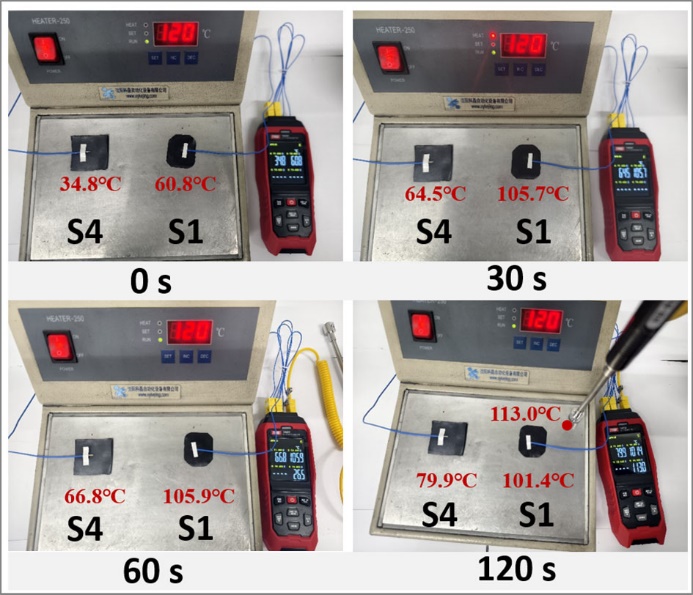


**Fig. S32** The actual surface temperatures of S1 and S4 measured by the thermocouple

**
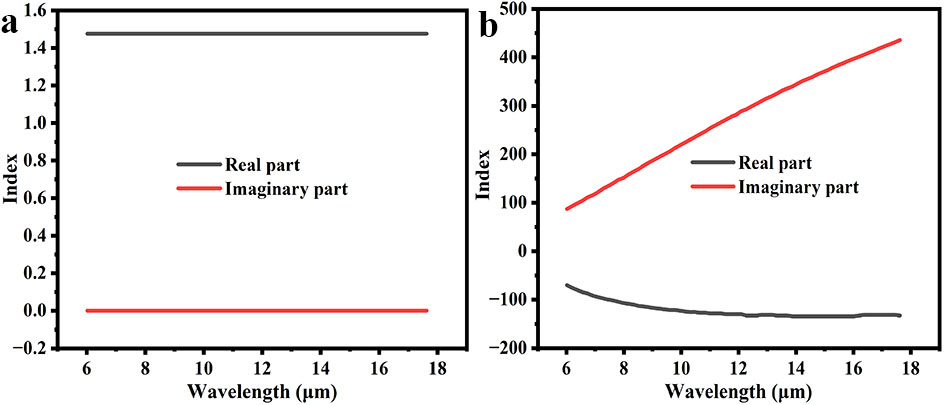
**

**Fig. S33** Optical parameters of (**a**) dielectric elastomers (DE) and (**b**) planar MXene film


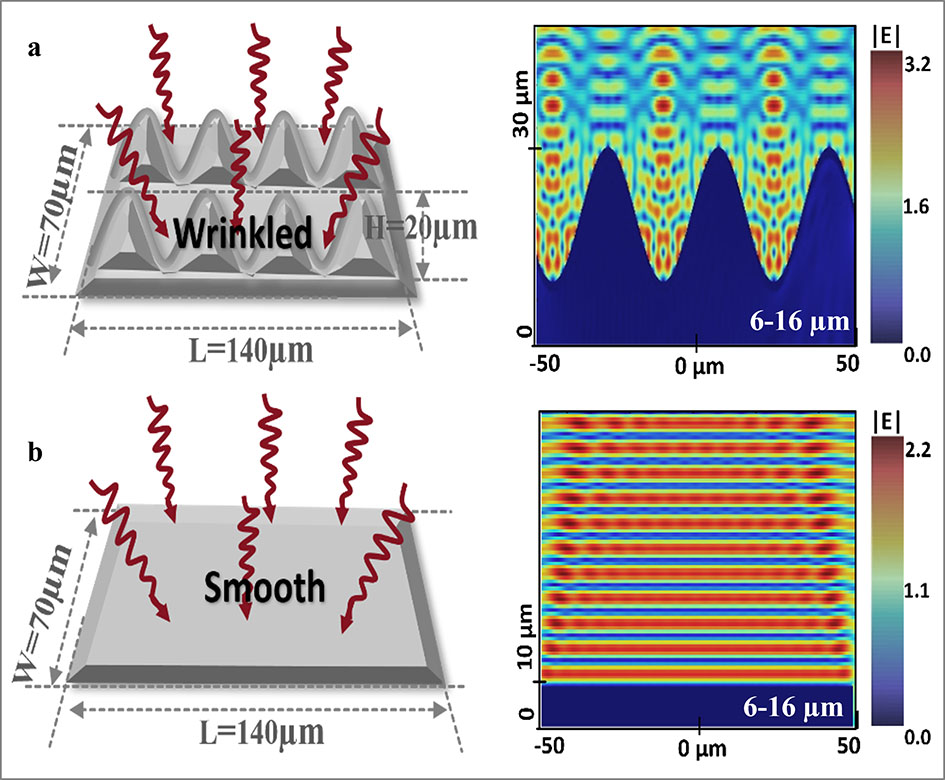


**Fig. S34** Optical simulation models and Electric field distributions (6-16 µm) of **a** wrinkled and **b** smooth MCFs


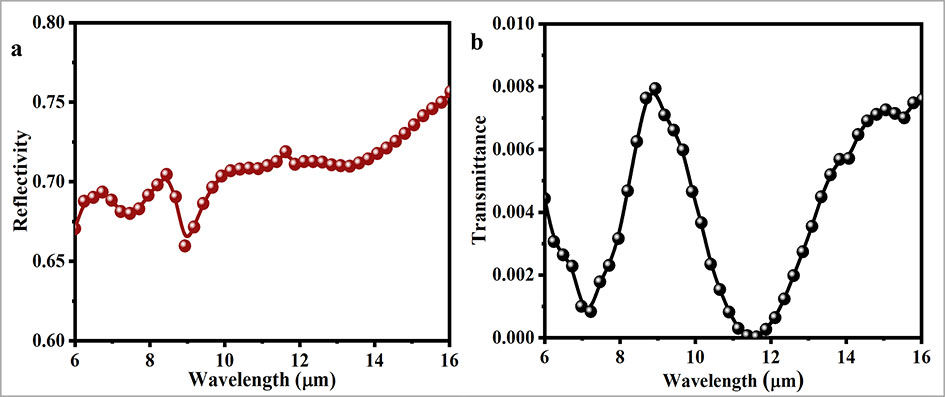


**Fig. S35** **a, b** Reflectivity and transmittance of wrinkled MCFs optical simulations

**Supplementary Videos**

**Video S1** Response time of the flexible wrinkled MCF attracted to magnet

**Video S2** The thermal camouflage effect of the smooth (left) and wrinkled (right) MCFs evolve over time (under oblique incidence)

**Video S3** The thermal camouflage effect of the S4-S6 MCFs evolve over time

**Video S4** Recovery time of damaged flexible wrinkle MCF

**Supplementary References**

[S1] X. Chai, D.M. Zhu, Y. Liu, Y.C. Qing, F. Luo et al., In-situ construction of Cr_2_O_3_@ATO hybrid pigment towards synergetic enhancement of visible light-infrared-radar compatible stealth. J. Colloid Interf. Sci. **645**, 570-579 (2023). <https://doi.org/10.1016/j.jcis.2023.04.175>

[S2] Q.Q. Huang, Z.D. Wang, Y. Zhao, Y. Wu, S.L. Tang et al., A graphene-based compatible flexible film with ultra-wideband microwave absorption and low infrared emissivity. Compos. Commun. **35**, 101349 (2022). <https://doi.org/10.1016/j.coco.2022.101349>

[S3] Y.P. Duan, B. Ma, L.X. Huang, X.R. Ma, M. Wang et al., Moth-eye-inspired gradient impedance microwave absorption materials with multiband compatible stealth characteristic. Adv. Mater. Technol. **8**, 2202172 (2023). <https://doi.org/10.1002/admt.202202172>

[S4] Z.M. An, Y.X. Huang, R.B. Zhang, High-temperature multispectral stealth metastructure from the microwave-infrared compatible design. Compos. Part B-Eng. **259**, 110737 (2023). <https://doi.org/10.1016/j.compositesb.2023.110737>

[S5] X.T. Chen, S.N. Guo, S.J. Tan, J.H. Ma, T. Xu et al., An environmentally friendly chitosan-derived VO_2_/carbon aerogel for radar infrared compatible stealth. Carbon **213**, 118313 (2023). <https://doi.org/10.1016/j.carbon.2023.118313>

[S6] Y. Xu, G. Wan, L. Ma, Y. Zhang, Y. Su et al., Indium tin oxide as a dual-band compatible stealth material with low infrared emissivity and strong microwave absorption. J. Mater. Chem. C **11**, 1754-1763 (2023). <https://doi.org/10.1039/d2tc04722e>

[S7] J. Wei, Y.B. Zhang, X.T. Li, J.W. Hui, H. Zhang et al., Multifunctional flower-like Ni particles/silicon carbide nanowires for infrared radar compatible stealth performance. J. Colloid Interf. Sci. **641**, 414-427 (2023). <https://doi.org/10.1016/j.jcis.2023.03.020>

[S8] C.H. Wan, R. Yan, S.S. Yu, X.Y. Wang, H.W. Tian et al., Novel ternary core-shell structure with microwave absorption properties and low infrared emissivity. Ceram. Int. **49**, 36516-36527 (2023). <https://doi.org/10.1016/j.ceramint.2023.08.335>

[S9] Q.Q. Huang, Y.Q. Zhang, S.J. Tan, Y. Wu, G.B. Ji et al., Multi-interfacial engineering in the hierarchical self-assembled micro-nano dielectric aerogel for wide-band absorption and low infrared emissivity. J. Colloid Interf. Sci. **649**, 76-85 (2023). <https://doi.org/10.1016/j.jcis.2023.06.076>

[S10] Q.Q. Huang, Y. Zhao, Y. Wu, M. Zhou, S.J. Tan et al., A dual-band transceiver with excellent heat insulation property for microwave absorption and low infrared emissivity compatibility. Chem. Eng. J. **446**, 137279 (2022). <https://doi.org/10.1016/j.cej.2022.137279>

[S11] M.T. Qiao, Y.R. Tian, J.X. Li, X.W. He, X.F. Lei et al., Core-shell Fe_3_O_4_@SnO_2_ nanochains toward the application of radar-infrared-visible compatible stealth. J. Colloid Interf. Sci. **609**, 330-340 (2022). <https://doi.org/10.1016/j.jcis.2021.12.012>

[S12] W.J. Ma, C.H. Tang, P. He, X.H. Wu, Z.K. Cui et al., Morphology-controlled fabrication strategy of hollow mesoporous carbon spheres@f-Fe_2_O_3_ for microwave absorption and infrared stealth. ACS Appl. Mater. Inter. **14**, 34985-34996 (2022). <https://doi.org/10.1021/acsami.2c08077>

[S13] P. He, W.J. Ma, J. Xu, Y.Z. Wang, Z.K. Cui et al., Hierarchical and orderly surface conductive networks in yolk-shell Fe_3_O_4_@C@Co/N-doped C microspheres for enhanced microwave absorption. Small **19**, 2302961 (2023). <https://doi.org/10.1002/smll.202302961>

[S14] Y. Cui, J.W. Ge, T. Ma, L. Liu, P.F. Ju et al., Enhanced electromagnetic wave absorption of Fe_3_O_4_@C derived from spindle-like MOF. Mater. Lett. **316**, 132060 (2022). <https://doi.org/10.1016/j.matlet.2022.132060>

[S15] B.D. Li, Z.H. Zeng, J. Qiao, Y.F. Yang, D.M. Xu et al., Hollow ZnO/Fe_3_O_4_@C nanofibers for efficient electromagnetic wave absorption. ACS Appl. Nano Mater. **5**, 11617-11626 (2022). <https://doi.org/10.1021/acsanm.2c02616>

[S16] W.H. Gu, J.Q. Sheng, Q.Q. Huang, G.H. Wang, J.B. Chen et al., Environmentally friendly and multifunctional shaddock peel‑based carbon aerogel for thermal‑insulation and microwave absorption. Nano-Micro Lett. **13**, 102 (2021). <https://doi.org/10.1007/s40820-021-00635->1

[S17] X.H. Wang, Y. Yuan, X.X. Sun, R. Qiang, Y.C. Xu et al., Lightweight, flexible, and thermal insulating carbon/SiO_2_@CNTs composite aerogel for high-efficiency microwave absorption. Small **20**, 2311657 (2024). <https://doi.org/10.1002/smll.202311657>

[S18] W.H. Gu, A.L. Xia, C.G. Jin, H.Y. Zhang, H.L. Li et al., An ultralight, eco-friendly 3D porous carbon aerogel derived from cotton nano-cellulose for infrared stealth and microwave absorption. Carbon **229**, 119565 (2024). <https://doi.org/10.1016/j.carbon.2024.119565>

[S19] X.K. Tian, H.J. Zhang, H.H. Li, K.X. Yang, Z.T. Xu et al., Multifunctional bacterial cellulose‑derived carbon hybrid aerogel for ultrabroad microwave absorption and thermal insulation. J. Colloid Interf. Sci. **677**, 804-815 (2025). <https://doi.org/10.1016/j.jcis.2024.08.120>

[S20] P.P. Zhou, S.Y Zhang, W. Zhou, C.C. Sun, C.Y. Hu et al., Hollow engineering of Co/N-doped C@carbon aerogel with hierarchical structure boosting interfacial polarization for ultra-thin microwave absorption and thermal insulation. Chem. Eng. J. **495**, 153561 (2024). <https://doi.org/10.1016/j.cej.2024.153561>

[S21] F.S. Wu, P.Y. Hu, F.Y. Hu, Z.H. Tian, J.W. Tang et al., Multifunctional MXene/C aerogels for enhanced microwave absorption and thermal insulation. Nano-Micro Lett. **15**, 194 (2023). <https://doi.org/10.1007/s40820-023-01158->7

[S22] Z.C. Zhang, Q.Y. Wang, Z.F. Li, Z. Zhou, X.Y. Xie et al., A skin-beyond multifrequency camouflage system with self-adaptive discoloration and radar-infrared stealth. Chem. Eng. J. **494**, 152867 (2024). <https://doi.org/10.1016/j.cej.2024.152867>

[S23]S.P. Shao, S.Z Xing, K. Bi, T.T. Zhao, H. Wang et al., Fabrication of graphene/polyimide/Co-N-C aerogel with reinforced electromagnetic losses and broadband absorption for highly efficient microwave absorption and thermal insulation. Chem. Eng. J. **494**, 152976 (2024). <https://doi.org/10.1016/j.cej.2024.152976>
